# High‐Conductivity, Self‐Healing, and Adhesive Ionic Hydrogels for Health Monitoring and Human‐Machine Interactions Under Extreme Cold Conditions

**DOI:** 10.1002/advs.202412726

**Published:** 2025-01-28

**Authors:** Fei Han, Shumeng Chen, Fei Wang, Mei Liu, Jiahui Li, Hao Liu, Yanshen Yang, Haoqing Zhang, Dong Liu, Rongyan He, Wentao Cao, Xiaochuan Qin, Feng Xu

**Affiliations:** ^1^ The Key Laboratory of Biomedical Information Engineering of Ministry of Education School of Life Science and Technology Xi'an Jiaotong University Xi'an 710049 P. R. China; ^2^ Bioinspired Engineering and Biomechanics Center (BEBC) Xi'an Jiaotong University Xi'an 710049 P. R. China; ^3^ School of Chemical Engineering and Technology Xi'an Jiaotong University No. 28, Xianning West Road Xi'an Shaanxi 710049 P. R. China; ^4^ Guangxi Key Laboratory of Special Biomedicine School of Medicine Guangxi University Nanning 530004 P. R. China

**Keywords:** health monitoring, human‐machine interactions, ionic conductive hydrogels, multifunctional wearable sensors, long‐term stability, self‐healing

## Abstract

Ionic conductive hydrogels (ICHs) are emerging as key materials for advanced human‐machine interactions and health monitoring systems due to their unique combination of flexibility, biocompatibility, and electrical conductivity. However, a major challenge remains in developing ICHs that simultaneously exhibit high ionic conductivity, self‐healing, and strong adhesion, particularly under extreme low‐temperature conditions. In this study, a novel ICH composed of sulfobetaine methacrylate, methacrylic acid, TEMPO‐oxidized cellulose nanofibers, sodium alginate, and lithium chloride is presented. The hydrogel is designed with a hydrogen‐bonded and chemically crosslinked network, achieving excellent conductivity (0.49 ± 0.05 S m^−1^), adhesion (36.73 ± 2.28 kPa), and self‐healing capacity even at −80 °C. Furthermore, the ICHs maintain functionality for over 45 days, showcasing outstanding anti‐freezing properties. This material demonstrates significant potential for non‐invasive, continuous health monitoring, adhering conformally to the skin without signal crosstalk, and enabling real‐time, high‐fidelity signal transmission in human‐machine interactions under cryogenic conditions. These ICHs offer transformative potential for the next generation of multimodal sensors, broadening application possibilities in harsh environments, including extreme weather and outer space.

## Graphical abstract

This study presents an ionic conductive hydrogel (ICH) with excellent conductivity, self‐healing, self‐adhesion, and long‐term stability under extreme cold conditions. It shows significant potential for non‐invasive, continuous health monitoring, conformally adhering to skin without signal crosstalk, enabling real‐time, high‐fidelity signal transmission in human‐machine interactions. It has huge potential for wearable sensors in harsh conditions.

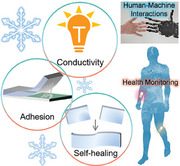

## 1 Introduction

Recently, flexible and wearable conductive hydrogels have attracted significant attention due to their potential applications in health monitoring, human‐machine interactions, and wearable electronics.^[^ ^1^ ^]^ Human‐machine interactions, which facilitate communication between humans and electronic systems, are increasingly important in areas like biosensing, electronic skin, and soft robotics.^[^ ^2^ ^]^ Among various conductive hydrogels, ionic conductive hydrogels (ICHs) have gained particular interest due to their unique combination of biocompatibility, transparency, and mechanical properties that closely mimic those of biological tissues.^[^ ^3^ ^]^ In addition, ICHs exhibit superior ionic conductivity, making them highly effective in maintaining the fidelity of electrical signals in dynamic, biologically relevant environments.^[^ ^3c^ ^]^ These properties make ICHs especially well‐suited for applications in artificial skin, soft robotics, and flexible electronics, where reliable and adaptive human‐machine interactions are crucial.^[^ ^2c^ ^]^ The ability of ICHs to mimic biological tissues and their responsiveness to environmental stimuli also positions them as promising candidates for next‐generation health monitoring systems, offering new possibilities for continuous, real‐time diagnostics in wearable devices.^[^ ^2a^ ^]^ As such, ICHs represent a key material in advancing both human‐machine interfaces and the development of more efficient and responsive health monitoring technologies.^[^ ^4^ ^]^

Despite their remarkable properties, existing ICHs face critical challenges that hinder their widespread application, particularly in wearable sensors and flexible electronics. One of the foremost limitations is their diminished performance under extreme temperatures. At high temperatures, the evaporation of water from the hydrogel matrix can lead to dehydration, while at low temperatures, the hydrogel network tends to freeze, both of which significantly impair conductivity and mechanical integrity.^[^ ^5^ ^]^ Moreover, prolonged exposure to open environments often results in water loss, causing shrinkage and a loss of function.^[^ ^6^ ^]^ In addition, the soft, flexible nature of ICHs leaves them vulnerable to mechanical damage—friction, tearing, compression, and torsion. All contribute to the degradation of hydrogel stability, particularly in harsh conditions such as cold climates or high‐latitude regions.^[^ ^7^ ^]^ These limitations severely restrict the usability and durability of ICHs in real‐world applications.

To tackle these challenges, several strategies have been employed to improve the environmental tolerance of ICHs.^[^ ^8^ ^]^ One method involves the incorporation of anti‐freezing agents, including organic solvents like ethylene glycol and glycerol, ionic liquids such as [EMIM]Cl and [BMIM]Cl, or inorganic salts like ZnCl_2_, CaCl_2_, and NaCl, to the hydrogel matrix, effectively lowering the freezing point of water.^[^ ^9^ ^]^ While these approaches have yielded some success, most solutions fall short of providing the multi‐functional characteristics needed, particularly high conductivity, self‐adhesion, and self‐healing under extreme low‐temperature conditions.^[^ ^10^ ^]^ This gap in performance highlights the urgent needs for ICHs that not only resist freezing but also maintain their functional properties in extreme environments. Polyzwitterions, a class of charged polymers bearing both positive and negative charges, have attracted significant attention due to their unique properties, including enhanced water retention capacity, adhesion, and conductivity.^[^ ^11^ ^]^ These advantages make polyzwitterions particularly appealing for use in ICHs. However, despite their promising characteristics, the “salt‐like structure” of polyzwitterionic hydrogels often leads to brittleness, limiting their mechanical robustness under stressful conditions.^[^ ^12^ ^]^

To address these challenges, a common strategy involves constructing double network hydrogels, where natural materials such as gelatin, agar, chitin, and sodium alginate serve as the first network, providing the hydrophilicity and biocompatibility needed for ionic conductivity.^[^ ^13^ ^]^ However, these natural materials alone exhibit fragility and poor mechanical properties.^[^ ^8b^ ^]^ The second network typically consists of synthetic polymers, including polyacrylic acid (PAA), polyacrylamide (PAAm), and polyvinyl alcohol (PVA), which are designed to enhance toughness and mechanical stability.^[^ ^2a^ ^]^ While such double networks can improve the mechanical strength, they still fail to addressing the critical issue of maintaining ionic conductivity and structural stability under extreme environmental conditions, particularly in cold or frozen environments. Besides, recent developments have aimed to introduce cellulose nanofibers (CNFs) as reinforcing agents for improving the mechanical strength of ICHs because of their high surface area, hydrophilicity, and biocompatibility.^[^ ^8c,d^ ^]^ The hierarchical fibrous structure of CNFs facilitates the formation of a hydrophilic interconnected network, which not only enhances water retention but also provides stable channels for ion transport, thereby improving the ionic conductivity.^[^ ^14^ ^]^ However, while CNFs have shown promise in improving the mechanical properties of hydrogels, they still struggle to provide long‐term stability and functional integrity in extreme low‐temperature conditions.

Existing studies, such as the work by Zhang et al., have demonstrated that ICHs can achieve self‐healing properties, high conductivity, and self‐adhesion simultaneously at room temperature.^[^ ^15^ ^]^ To extend the functionality of ICHs in cold environments, Guo et al. incorporated these materials into touch panels, though their performance was limited by a narrow operating temperature range.^[^ ^16^ ^]^ In contrast, Fu et al. fabricated ICHs that exhibited high conductivity and self‐adhesion but lacked self‐healing properties, highlighting a trade‐off between different functional attributes in existing materials.^[^ ^12^ ^]^ To overcome these challenges, we introduced a multifunctional ICH that features self‐healing properties, high conductivity, and self‐adhesion ability, designed for use in extreme cold conditions. In these systems, sulfobetaine methacrylate (SBMA) and methacrylic acid (MAA) build up the polymeric skeleton based on hydrogen bonds and electrostatic interactions, while the TEMPO‐oxidized cellulose nanofibers (CNF), sodium alginate (SA), and lithium chloride (LiCl) are embedded in this skeleton. The electrostatic interactions between zwitterionic groups (the electrostatic interactions between zwitterionic groups (‐N^+^(CH_3_)_3_ or ‐SO_3_ in SBMA)^[^ ^11^ ^]^ and Li^+^ ions contribute to the high ionic conductivity exhibited by these hydrogels, reaching 2.21 ± 0.35 S m^−1^ at room temperature and 0.49 ± 0.05 S m^−1^ at −80 °C. Furthermore, these effects underpin the robust adhesion of 40.52 ± 2.66 kPa at room temperature and 36.73 ± 2.28 kPa at −80 °C owing to the dipole‐dipole interactions between the hydrogel and the substrates.^[^ ^17^ ^]^ In addition, the introduction of LiCl enhances the hydration of Li^+^ ions, which transforms free water into a bound state. This disrupts the directional arrangement of hydrogen bonds within the hydrogel matrix under room temperature and extreme cold temperatures. Besides, its functionality is still maintained over 45 days because of its anti‐freezing and long‐term stability. This multi‐functional ICH serves as a versatile material platform for heath monitoring and human‐machine interactions at both normal and extreme low temperatures. These innovations have considerable potential to influence the creation of next‐generation wearable devices, particularly in biomedical applications such as continuous health monitoring, smart prosthetics, and human‐machine interfaces, where environmental durability is paramount. This research bridges a critical gap in the field of ionic hydrogels, offering a versatile material platform for use in extreme conditions, and represents an important step forward in the design of bioinspired, functional biomaterials for real‐world applications.

## 2 Results and Discussion

### 2.1 Design of ICHs for Human‐Machine Interactions and Health Monitoring

**Figure 1:**
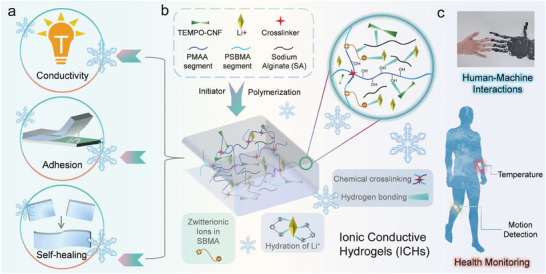
Schematic Illustration of the synthesis, properties, and applications of ICHs. a) Properties of ICHs under extreme low temperatures including high conductivity, adhesion, and self‐healing. b) Design principle, fabrication process, molecular structures, and interactions of ICHs under extreme low temperatures. c) ICHs for human‐machine interactions and health monitoring.

To synthesize ionic conductive hydrogels (ICHs) with anti‐freezing properties, high conductivity, and long‐term stability for human‐machine interactions and health monitoring, we incorporated LiCl, TEMPO‐CNFs, and SA into a polymer matrix composed of MAA, SBMA, APS, and MBA, as shown in **Figure**  **1**. We performed the synthesis via free radical polymerization, achieving successful formation of the ICHs. The addition of SA provides more dynamic cross‐linking sites, significantly enhancing the structural integrity of the hydrogels, as evidenced in Figure S1a (Supporting Information).^[^ ^18^ ^]^ Additionally, the advantageous properties of TEMPO‐CNFs can improve the ionic conductivity of these ICHs, as their hierarchical porous structures offer increased surface area. The carboxyl groups on the TEMPO‐CNFs can attract counterions and create more hopping sites for ion transfer.^[^ ^3a^ ^]^ To further quantify the impact of TEMPO‐CNFs on ionic conductivity, we evaluated the conductivity at varying concentrations of CNFs in the ICHs. As shown in Figure S1b (Supporting Information), the ionic conductivity of the hydrogels increased with higher CNF content. More importantly, the self‐healing ability of the ICHs can be fine‐tuned by adjusting the LiCl concentration (Figure S1c, Supporting Information). When LiCl is introduced, the strong hydration of Li^+^ converts free water into a bound state, disrupting the directional arrangement of hydrogen bonds. This transformation activates hydrogen bonds, allowing for efficient reversible breaking and reforming, thereby enhancing the self‐healing properties.^[^ ^3c^ ^]^ The combination of SA and TEMPO‐CNF plays a crucial role in both the successful fabrication and the mechanical performance of the ICHs. Our experiments show that SA is essential for the formation of the hydrogel matrix. Specifically, the hydrogel cannot be successfully fabricated without SA, as it is necessary for the network structure and the overall stability of the hydrogel. According to Figure S1d (Supporting Information), rheological measurements revealed that the inclusion of TEMPO‐CNF significantly enhances the ICHs mechanical properties, particularly its shear storage modulus, without substantially altering its rheological properties in the absence of SA. Thus, the synergistic combination of SA and TEMPO‐CNF enables the hydrogel to maintain both its structural integrity and enhanced mechanical properties, while also ensuring that its ionic conductivity is preserved for effective performance in human‐machine interactions and health monitoring.

### 2.2 Self‐Healing, Adhesion, Electrical Properties, and Long‐Term Stability of ICHs under Room Temperature and Extreme Cold Temperatures

**Figure 2:**
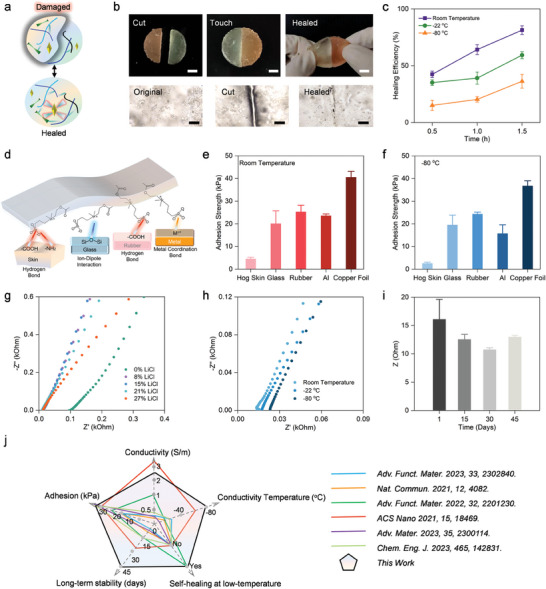
Self‐healing, adhesion, and electrical properties of ICHs. a) Self‐healing mechanism of ICHs. b) Separated pieces of ICHs merging into one sample after self‐healing and under stretching. Scale bar: 1 cm (upper). Self‐healing process of one damaged ICH observed through the optical microscope. Scale bar: 50 µm (lower). c) Self‐healing efficiency (%) of ICHs healed at room temperature, ‐22 and −80 °C for 0.5, 1.0, 1.5 h. d) Adhesion mechanism of ICHs on hog skin, glass, rubber, and metal. The lap shear tests of ICHs adhesion properties at e) room temperature and f) −80 °C on various substrates (hog skin, glass, rubber, Al, and copper foil). g) EIS plot of ICHs with different content of LiCl. h) Conductivity of ICHs under different temperatures (room temperature, −22 and − 80 °C). i) The long term conductive stability after 45 days. j) Comparison of comprehensive performance with other reported materials in the literature. Error bars: standard deviation (n = 3, n is the sample size for each group).

To gain comprehensive insights into the autonomous self‐healing behavior, **Figure**  **2**a illustrated that the rapid recovery of the structure‐network and functions are attributed to the reversible interactions within the hydrogel.^[^ ^3f^ ^]^ We produced severe mechanical damage by completely separating the ICH into two pieces. We then observed the healing process as the damaged segments of the hydrogel were gently brought together. Notably, Figure  2b depicted the healing process at the damaged surface visually, revealing the merging of the notch within 30 min of healing. To enhance visibility, two ICHs were dyed in distinct colors. Despite the visible boundary after 30 min, the conformation of the polymer network remained unchanged by signifying the consistency of the healing process. The evaluation of healing efficiency was conducted for all samples in Figure  2c, Figure S2, and Table S1 (Supporting Information) by using a key parameter that quantifies the recovery of mechanical properties, defined as (ε_h_/ε_o_) × 100%, where ε_h_ represents the healed strain and ε_o_ indicates the original strain.^[^ ^19^ ^]^ Remarkably, the mechanical properties of the ICHs were maintained by demonstrating similar tensile stress‐strain curves. At room temperature, the ICHs exhibited a 42% recovery within 30 min, escalating to 81% after 1.5 h of healing.

To ensure its functionality at extreme cold conditions, we pre‐evaluated the anti‐freezing property of inorganic salt LiCl. It behaved with outstanding anti‐freezing performance after quite a long time at −80 °C (Figure S3a, Supporting Information). To gain a deeper understanding of the anti‐freezing properties of the ICHs, differential scanning calorimetry (DSC) results (Figure S3b, Supporting Information) revealed that the ICHs displayed an exothermic peak at −8.7 °C during the cooling process from 20 to −80 °C when the LiCl content reached 8%, indicating that plenty of ice crystals were formed.^[^ ^20^ ^]^ However, the freezing point cannot be detected when the LiCl content increased to 21% and 27%, which manifested that LiCl played a crucial role in decreasing the freezing point of ICHs. This unprecedented tolerance of ICHs at extreme low temperatures is attributed to the robust interaction between LiCl and water, which prevents the aggregation of water molecules and inhibits ice crystal formation.^[^ ^21^ ^]^ To further investigate the ion dynamics of our hydrogel at sub‐zero temperatures, we conducted dielectric relaxation spectroscopy (DRS) measurements (Figure S3c, Supporting Information). These measurements revealed that the hydrogel exhibits a very high dielectric constant, even at −80 °C, indicating that the material maintains excellent ionic mobility in extreme cold conditions.^[^ ^22^ ^]^ This high dielectric constant and dielectric loss are crucial for explaining the retention of ionic conductivity, as it suggests that the ionic environment within the hydrogel matrix remains stable, ensuring continued conductivity at low temperatures. The DRS data correlate well with our conductivity measurements, further confirming that the ionic conductivity is sustained in extremely cold environments. This underscores the unique capability of the ICHs to function effectively as a wearable sensor or component for human‐machine interactions, even in low‐temperature settings. Maintaining its self‐healing ability even under extreme low temperatures, ICHs exhibited recovery efficiencies of 81%, 59%, and 46% after 1.5 hours of healing at room temperature, −22 °C, and −80 °C, respectively (Figure  2c). However, healing process decelerated due to limited diffusion of polymer segments and the reorganization of dynamic interactions under extreme low temperatures. As a result, extending the healing time effectively improved self‐healing efficiency.

The ICHs exhibit strong adhesive properties with a range of materials, including glass, aluminum (Al), copper foil, and rubber (Figure  2d). The outstanding adhesion of ICHs arises from the combined effects of chemical interactions and energy dissipation that occur during the peeling process.^[^ ^17b^ ^]^ The molecular mechanisms underlying the strong adhesion of our ICHs are primarily attributed to a combination of electrostatic interactions, hydrogen bonding, and ionic bonding within the hydrogel matrix. The zwitterionic sulfonate and quaternary ammonium groups in SBMA generate dipole‐dipole and ion‐dipole interactions with the surface of substrates, promoting strong adhesion.^[^ ^23^ ^]^ In addition, the hydrophilic nature of both SBMA and TEMPO‐CNF facilitates the formation of hydrogen bonds between the hydrogel and substrate surface, further strengthening the adhesion.^[^ ^24^ ^]^ The incorporation of Li^+^ ions in the hydrogel matrix results in ionic bond, which not only enhances their ionic conductivity but also reinforces its adhesion properties.^[^ ^24^ ^]^ This ionic network stabilizes their structure and prevents adhesion failure under external stress. These features, coupled with the self‐healing ability and anti‐freezing properties of our material, allow for enhanced adhesion performance compared to other hydrogels that do not exhibit these combined characteristics. Concurrently, the dynamic metal coordination bonds and hydrogen bonds within ICHs offer a dissipation mechanism that demands extra energy to propagate interfacial cracks.^[^ ^25^ ^]^ The strong adhesion of our ICHs to both metal and rubber substrates is primarily driven by a combination of physical and chemical bonding. The zwitterionic groups in SBMA interact with the substrate surfaces through electrostatic interactions, particularly with hydroxyl groups or reactive sites on the metal and rubber surfaces.^[^ ^24^ ^]^ In addition, hydrogen bonding between the hydrogel matrix and the substrates contributes to the overall adhesion strength, while Van der Waals forces further reinforce the bond.^[^ ^24^ ^]^ Adhesion strength, evaluated through lap shear tests on assemblies with ICHs sandwiched between substrate pairs (Figure S4a, Supporting Information), was measured as 20.08 ± 5.66 kPa for glass, 25.26 ± 2.95 kPa for rubber, 23.55 ± 0.80 kPa for Al, and 40.52 ± 2.66 kPa for copper foil (Figure  2e). The maximum adhesion strength reflects the interfacial adhesion capacity at the point of failure, serving as an indicator of the interface's overall adhesive strength.^[^ ^5b^ ^]^ The remarkable resistance to shear testing can be attributed to the cohesive forces within ICHs, which dissipate energy during the peeling process, allowing the hydrogels to endure significant peeling forces.^[^ ^26^ ^]^

Adhesion properties at extreme low temperatures (Figure  2f) revealed stable interface connections between ICHs and various substrates, maintaining 19.49 ± 4.38 kPa for glass, 24.30 ± 0.87 kPa for rubber, 15.71 ± 3.88 kPa for Al, and 36.73 ± 2.28 kPa for copper foil at −80 °C. These adhesion mechanisms remain robust even at low temperatures, where the hydration shell around Li^+^ ions plays a critical role in stabilizing the ionic interactions that facilitate adhesion.^[^ ^16^, ^27^ ^]^ This hydration shell prevents the hydrogel from freezing and maintains its adhesive properties by ensuring the strength of the molecular interactions, including hydrogen bonding, ionic bonding, and electrostatic interactions between the hydrogel and substrate even at temperatures as low as −80 °C.^[^ ^3^, ^28^ ^]^ Besides, the LiCl could effectively lower the freezing point of the system, maintaining the soft and flexible nature of ICHs even at extremely low temperatures.^[^ ^29^ ^]^ These features combine to ensure that our ICHs exhibit robust and stable adhesion to a variety of substrates even under extreme low‐temperature conditions. When compared with other reported ICHs, our ICHs exhibit superior adhesion performance, particularly under extreme conditions. For instance, at room temperature, the adhesion strength of our ICHs is ≈40.52 ± 2.66 kPa, which is higher than that reported for zwitterionic ICHs by Yu et al. (20.9 kPa)^[^ ^1b^ ^]^ and cellulose‐based hydrogels by Zong et al. (35 kPa).^[^ ^30^ ^]^ At extreme low temperatures, our ICHs maintain strong adhesion (36.73 ± 2.28 kPa at −80 °C), while other ICHs, such as those developed by Fu et al., show a significant decrease in adhesion strength (60% reduction at −20 °C).^[^ ^31^ ^]^ Based on this property, this characteristic is advantageous for applications involving ICHs in cryogenic environments. Additionally, as depicted in Figure S4b,c (Supporting Information), ICHs maintained intimate contact with hog skin even during twisting, demonstrating their potential for human motion sensors with desirable adhesion strength at 4.88 ± 2.33 kPa. The dependable adhesion of ICHs demonstrated impressive repeatability and stability, retaining 50% to 80% of the original adhesion strength after five consecutive peeling cycles across various substrates (Figure S4d, Supporting Information). The slight reduction in adhesion strength may be due to damage or contamination from dirt, while the saturation of contaminants in subsequent cycles helped limit further decreases in interfacial adhesiveness.^[^ ^32^ ^]^

To explore its ionic conductivity, Figures 2g and S5a,b (Supporting Information) present the Nyquist plot of ICHs equilibrated in varying concentrations of LiCl. The ICH‐0% exhibits relatively low conductivity, as the gel contains only a limited number of free ions due to the restricted ionization of the carboxyl groups in TEMPO‐CNF (Figure S1b, Supporting Information).^[^ ^33^ ^]^ The addition of LiCl induces a shift in impedance toward lower values, indicating an augmented ionic conductivity. Notably, there is a sharp increase in ionic conductivity for ICH‐15%, highlighting the enhanced performance resulting from the addition of salt ions. However, it is crucial to recognize that increasing the salt concentration gradually reduces the ionic conductivity of the gel, as the contraction of the gel network restricts ion mobility.^[^ ^34^ ^]^ Figure S6a (Supporting Information) further demonstrated small‐angle X‐ray scattering (SAXS) results, indicating that the system exhibited a more evenly packed structure at 15% LiCl. However, increasing LiCl to 21% led to the collapse of the matrix structure, resulting in the emergence of unevenly packed structures. This transformation was also supported by a significant increase in intensity and a shift in the scattering spectra within the low q regime. The influence of LiCl on the mechanical properties of ICHs was further investigated in Figure S6b (Supporting Information). The ICH‐0% hydrogel exhibits desirable strain tolerance due to robust crosslinking among polyzwitterions, SA, and TEMPO‐CNF. An increase in LiCl content (from 0 to 8 wt.%) leads to a reduction in mechanical strength due to the anti‐polyelectrolyte effect induced by LiCl, weakening the aforementioned interactions.^[^ ^35^ ^]^ However, a further increase in LiCl content significantly optimizes the mechanical properties. This enhancement is attributed to the release of more dynamic hydrogen bonds from the polymer skeleton, dissipating larger energy and homogenizing the network.^[^ ^36^ ^]^ Consequently, ICHs become more resilient, allowing deformation without fracture. The anti‐freezing property stands out as a crucial attribute for ICHs, particularly in applications such as arctic conditions and winter sports.

To further assess the anti‐freezing properties, we measured the ionic conductivity of the hydrogels at room temperature, −20 °C, and −80 °C (Figure  2h; Figure S5c,d, Supporting Information). This remarkable anti‐freezing effect is attributed to the presence of LiCl and its interaction with water, wherein the hydration of LiCl salt prevents ice formation at sub‐zero temperatures, establishing an effective anti‐freezing mechanism. Based on the electrochemical impedance spectroscopy (EIS) spectra, the impedance increases at reduced temperatures due to restricted ion mobility,^[^ ^37^ ^]^ resulting in a decline in ionic conductivity from 2.21 ± 0.35 S m^−1^ at room temperature to 1.09 ± 0.43 S m^−1^ at −22 °C and 0.49 ± 0.05 S m^−1^ at −80 °C. It is noteworthy that the ionic conductivity at −80 °C remains at 0.49 ± 0.05 S m^−1^, which is higher than that of ICH‐0% at room temperature. To assess long‐term stability, ICHs were stored at room temperature for 45 days, revealing sustained excellent conductivity (Figure  2i; Figure S5e,f, Supporting Information). After this period, the resistance of ICH shows only slight changes, maintaining at 12.97 ± 0.29 ohm. The weight of ICHs retains their original state with minimal loss (Figure S6c, Supporting Information). Even after 45 days, ICHs exhibit satisfactory mechanical properties and adhesion strength (Figure S6d,e, Supporting Information), mainly attributed to robust hydrogen bonds between LiCl and water.^[^ ^28^ ^]^ To evaluate the long‐term stability of ICH adhesion in extremely cold environments, we conducted an experiment in which the ICHs were attached to hog skin for a period of 45 days at −80 °C (Figure S6f, Supporting Information). The results demonstrated that the ICHs retained excellent adhesive properties without significant degradation even after prolonged exposure to such low temperatures. This indicates that the interfacial adhesion of the ICHs is not compromised by environmental icing or extended exposure to extreme cold. These findings underscore the suitability of ICHs for use in wearable monitoring systems, particularly for applications in low‐temperature environments, where the stability of adhesion over time is crucial. In conclusion, ICHs demonstrate preferable properties for long‐term use by maintaining their initial state. Comparative analysis with counterparts from the literature showcases the significantly improved collective performance of our ICHs in terms of adhesion, conductivity, conductivity temperature, self‐healing at low temperatures, and long‐term stability (Figure  2j).^[^ ^12^, ^14^, ^15^, ^16^, ^30^, ^38^ ^]^

### 2.3 Design Principle and Information Transmission Capacity of ICHs and their Application in Human‐Machine Interactions

**Figure 3:**
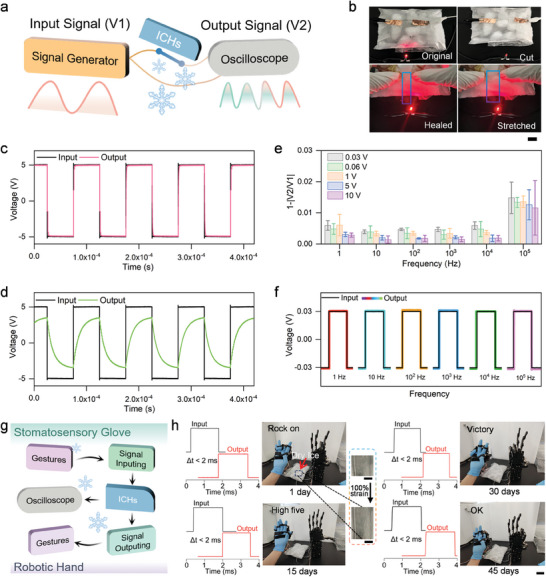
Design principle and information transmission capacity of ICHs and their application in human‐machine interactions a) Design principle of ICHs. b) The electrical self‐healing capacity of ICH at extreme low temperatures. Scale bar: 2 cm. Comparison of signal transmission between c) ICHs and (d) hydrogels without adhesion. e) 1‐|V2/V1| varied with frequency and amplitude of voltage pulses. f) Signal curves recorded in ±0.03 V under frequency from 1 Hz to 10^5^ Hz. Error bars: standard deviation (n = 3, n is the sample size for each group). g) Simple circuit diagram of stomatosensory glove and robotic hand connected by ICHs. h) Stomatosensory glove and robotic hand connected by ICHs demonstrating different gestures at extreme low temperatures during 45 days. Scale bar: 2 cm. A local cryogenic environment was created by dry ice. The input and output signals of ICHs demonstrate only 2 ms delay under 100% deformation at extreme low temperatures. The system maintained uninterrupted synchronization under 100% stretching in extreme cold conditions even after 45 days (Inset: The 100% deformation of ICHs on dry ice. Scale bar: 0.5 cm).

To confirm its information transmission capability, **Figure**  **3** demonstrates the compatibility of ICHs with flexible electronic systems. They are essential for connecting corresponding functional devices. In addressing the challenges posed by the hardness and brittleness of traditional metal wires, organic conductive materials have been extensively explored.^[^ ^39^ ^]^ However, the intrinsic resistance and strain sensitivity of stretchable conductors often result in significant driving voltage, heat loss, and unstable signal transmission performance.^[^ ^40^ ^]^ Given their collective performances, ICHs emerge as a promising alternative for information transmission. We used a signal generator as the input source and an oscilloscope to analyze the transmission curves both before and after the signal propagated through the hydrogel (Figure  3a). The ICHs display excellent transmission performance, maintaining high‐fidelity signal output even after mechanical damage under extreme cold conditions (Figure  3b). We tested their electrical healing ability by cutting the hydrogels into two parts and then observing their recovery. Upon reconnecting the two parts, the red LED immediately regains brightness, demonstrating the rapid restoration of conductivity. After 30 minutes of autonomous healing without external stimuli or added substances, the ICHs fully recover their mechanical properties.

We further evaluated the signal fidelity by comparing the input (V1) and output (V2) signals. Unlike other hydrogels that lack adhesion, there were minimal phase and shape changes in V2 compared to V1 (Figure  3c,d). Notably, the value of 1‐(V2/V1) remained between 0.001 and 0.15 as the frequency varied from 1 Hz to 1 MHz, and the voltage amplitude varied from 60 mV to 20 V, underscoring the excellent information transmission fidelity of ICHs (Figure  3c–f; Figure S7, Supporting Information). The ICHs also exhibit high‐fidelity transmission for various voltage signals, including triangular and sine waves (Figures S8–S10, Supporting Information). In our study, we explored the performance of the ICHs at high frequencies (up to 10 MHz and beyond) to assess potential issues such as signal noise and phase shifting, which are critical factors in applications involving dynamic data transmission. The oscilloscope data in Figure S8d,e (Supporting Information) demonstrated that the ICHs exhibit minimal signal loss and no detectable phase shifting even at these elevated frequencies. This indicates that the hydrogels maintain high‐fidelity signal transmission in high‐frequency regimes, which is essential for applications in wearable electronics and health monitoring systems that require dynamic and continuous data transmission.^[^ ^41^ ^]^ The results further suggest that the ionic conductivity of the hydrogel is stable across a wide frequency range, contributing to robust performance without significant signal degradation or noise interference. To assess real‐time message transmission performance with integrated ICHs, we incorporated them into a synchronous bionic robotic hand system (Figure  3g; Figure S11, Supporting Information). In this setup, a somatosensory glove served as the signal input device, the robotic hand acted as the signal output, and an oscilloscope was connected to both ends for monitoring. To simulate a cryogenic environment, ICHs were applied to the surface of dry ice (−80 °C) during testing. We investigated the potential of ICHs in human‐machine interaction applications (Figure  3h). After the somatosensory glove detected hand movements, encoded the corresponding gesture signals, and transmitted integrated messages, the ICHs relayed these signals to the robotic hand, which decoded them and performed the appropriate actions. The oscilloscope recorded the input and output signals, revealing a time delay of only 2 ms under 100% stretching at the cryogenic temperature. The robotic hand's synchronous actions remained uninterrupted, demonstrating their excellent transmission stability. Even after 45 days, the system maintained uninterrupted synchronization under 100% stretching in extreme cold conditions.

### 2.4 Application of ICHs in Health Monitoring

**Figure 4:**
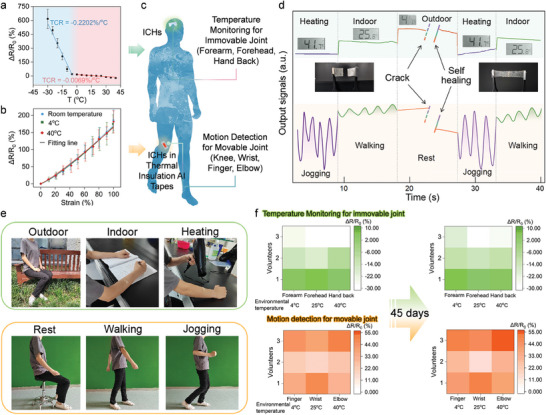
Application of ICHs in health monitoring. a) Relative resistance variation of ICHs temperature sensors from −30 to 40 °C. The slope of the linear fitting curves demonstrates the TCR value of ICHs for temperature monitoring. b) Relative resistance changes of ICHs in thermal insulation aluminium (Al) tapes under different environmental temperatures versus the applied strain over a strain of 0–100% for motion detection. c) ICHs could be directly attached on immovable joint for temperature monitoring independently without any interruption of body motion. ICHs in thermal insulation Al tapes could be attached on movable joint for motion detection independently without any interruption of environmental temperature. d) Output signals (temperature in green and motion in yellow) measured by ICHs and ICHs in thermal insulation Al tapes before fracture, upon fracture, and after self‐healing. e) Demonstration of ICHs attached to the immovable joint to detect temperature and ICHs in thermal insulation Al tapes to detect varying motion in different activities under different environmental temperatures. f) The comparison of ICHs and ICHs in thermal insulation Al tapes’ sensory network for detection of different volunteers’ temperature and body motion at 0 day and after 45 days. Error bars: standard deviation (n = 3, n is the sample size for each group).

To develop a straightforward solution for measuring skin peripheral temperature and motion, we utilized ICHs to evaluate both parameters (**Figure**  **4**).^[^ ^38^ ^]^ As shown in Figure  4a, the slope of the fitted curves indicates the temperature coefficient of resistance (TCR), which ranges from −0.2202 to −0.0069 × 100% °C^−1^ at ‐30 to −5 °C and 0 to 40 °C, respectively. This effect arises due to the impact of temperature variations on ion mobility within the hydrogel.^[^ ^42^ ^]^ Compared to the incorporation of ICHs without TEMPO‐CNF (Figure S12a, Supporting Information), one significant advantage of TEMPO‐CNFs is their ability to form hierarchical networks through hydrogen bonds and electrostatic interactions.^[^ ^20^ ^]^ The thermal vibrations of TEMPO‐CNFs activate electrons, allowing them to efficiently cross the potential polymer barrier as the temperature rises, which in turn induces resonant tunneling currents.^[^ ^9c^ ^]^ Additionally, the temperature sensing performance during long‐term exposure was assessed to evaluate stability, as shown in Figure S12b (Supporting Information). The TCR measurements taken after 45 days were comparable to those in the original state, indicating that both flexibility and sensing performance were well preserved. The slight difference between them was attributed to a small amount of water evaporation over 45 days. Furthermore, the instantaneous electrical response to temperature changes was measured to further assess the transient thermosensitive capacity (Figure S12c, Supporting Information). When a heat source is brought close, the resistance decreases sharply due to its negative temperature coefficient. Conversely, the resistance increases steadily when exposed to a cold source. Notably, the resistance recovers from heating much faster than it does from cooling. This occurs because a larger temperature difference between the heat source and the surrounding environment enhances thermal convection, resulting in a more pronounced and rapid change in resistance.^[^ ^10d^ ^]^ To investigate the effect of repeated thermal cycles on the performance of the ICHs, we conducted a series of heating and cooling cycles (Figure S12c, Supporting Information). The hydrogel demonstrated a slight degradation in its performance, primarily during the cooling process, which we attribute to structural reorganization and changes in ionic mobility.^[^ ^3f^ ^]^ Specifically, as the hydrogel cools, the ionic mobility and hydration of the hydrogel network may decrease, leading to a reduction in conductivity.^[^ ^38^ ^]^ Although these changes were minimal, they were more pronounced during cooling than during heating, indicating a slight thermal fatigue over multiple cycles. Despite this, the hydrogel retained a significant level of functionality, with only marginal changes in performance after several cycles.

To demonstrate strain‐sensing performance of ICHs, Figure  4b illustrated that the stretching deformation remains consistent across different environmental temperatures. This stability is achieved by encapsulating the ICHs in thermal insulation aluminum (Al) tapes for motion detection. As seen in Figure S12d–f (Supporting Information), the temperature of the ICHs remains unaffected by environmental temperatures after encapsulation. Based on this, Figure  4c demonstrates ICHs could be attached on immovable joint (forearm, forehead, hand back, etc.) for temperature monitoring independently, and ICHs could be encapsulated in thermal insulation Al tapes and attached on movable joint (knee, wrist, finger, elbow, etc.) for motion detection independently. This design effectively separates temperature monitoring and motion detection, preventing signal crosstalk and ensuring precise data acquisition during health monitoring. Considering that the stretchability of biological skin typically does not exceed 75%, these ICHs are well‐suited as skin‐attachable motion sensors for monitoring human movements.^[^ ^43^ ^]^ The decent sensitivity in this strain range renders them appropriate for diverse sensing applications scenarios.^[^ ^12^, ^16^, ^37^, ^44^ ^]^ As depicted in Figure S13a (Supporting Information), the relative resistance change to strain (ranging from 10–60%) displayed remarkable sensing repeatability during cyclic tension. In addition, these ICHs demonstrated highly stable and uniform resistance signals across different strain rates (Figure S13b, Supporting Information), highlighting their excellent dynamic responsiveness. Thanks to their remarkable sensitivity and recovery properties, these ICHs reacted within just 387 ms and promptly returned to their original state within 400 ms under 30% strain (Figure S13c, Supporting Information). The response time of wearable sensors, particularly those based on hydrogels, is governed by multiple factors, including ion mobility, the mechanical properties of the hydrogel matrix, and the interactions between the hydrogel and external stimuli.^[^ ^3c^ ^]^ In the case of our ICHs, the ionic conductivity plays a crucial role in determining the response time. The movement and redistribution of ions within the hydrogel network contribute to the delay in resistance change.^[^ ^3a^ ^]^ Specifically, the rate at which ions diffuse through the network, influenced by electrostatic interactions and hydration effects, can significantly slow down the response time.^[^ ^14^ ^]^

Furthermore, the interaction between the ions embedded in the hydrogel matrix can create this delay. The movement of ions such as Li^+^ or Na^+^ is affected by their interactions with the polymeric matrix and the associated hydration shell.^[^ ^3a^ ^]^ These interactions influence the overall response time. This hypothesis is supported by the results shown in Figure S13c (Supporting Information), where ICHs without LiCl or TEMPO‐CNF exhibit a shorter response time. This suggests that the presence of ions and their interactions within the hydrogel network contribute to the observed delay. In addition to ion interactions, the encapsulation of the hydrogel sensor in thermal insulation Al tapes, can introduce mechanical damping that further affects the response time. The insulation layer can slow down the transmission of external mechanical forces to the hydrogel matrix, which in turn delays the mechanical deformation and the subsequent change in resistance.^[^ ^45^ ^]^ This effect is also illustrated in Figure S13c (Supporting Information), where ICHs encapsulated in insulation layers exhibit a slightly longer response time compared to those without encapsulation, providing further evidence of the mechanical damping effect. Overall, both ion interactions within the hydrogel matrix and the encapsulation with thermal insulation materials contribute to the observed delay in the response time of the ICH‐based sensor. These factors highlight the complex interplay of material properties and environmental conditions that govern the performance of wearable sensors. Besides, the scanning electron microscopy (SEM) images (Figure S13d, Supporting Information) of the ICHs show that the TEMPO‐CNFs maintain an isotropic state, both in the original and stretched states. This isotropic distribution ensures that there is no significant anisotropic alignment of TEMPO‐CNFs under strain, which could potentially affect conductivity. This isotropic distribution of TEMPO‐CNFs contributes to the uniform conductivity observed in the ICHs.

To evaluate the electromechanical properties of ICHs, we characterized the normalized resistance change (ΔR/R_0_) of ICHs (10 × 2 mm) on VHB tape under uniaxial tensile strain.^[^ ^46^ ^]^ The electromechanical performance of ICHs remains stable over 2500 cycles for cyclic tensile strains up to 10% (Figure S14, Supporting Information). After cyclic stretching, ICHs preserved their structural integrity with no observable network disconnection within the hydrogel matrix. While a slight drift in resistance changes was observed due to mechanical hysteresis, there was no significant degradation in the resistance change signal.^[^ ^3a^ ^]^ Additionally, as shown in Figure S15 (Supporting Information), ICHs in thermal insulation Al tapes function effectively at different environmental temperatures. As the finger, wrist, or elbow gradually bent with step‐wise amplitude at varying environmental temperatures, the ΔR/R_0_ increased correspondingly and remained stable without significant noise fluctuations or hysteresis. Notably, no permanent deformation occurred. These results demonstrate the stable and durable strain sensitivity of the ICH sensor across different environmental conditions.

Figure  4d,e demonstrated that the skin temperatures and varying motions are effectively measured by ICHs without crosstalk. ICHs are attached on immovable joint for measuring body temperatures at low (outdoor), medium (indoor), and high temperature (heating) (depicted in the green part), which agree reasonably well with those from the **Figure**  **4a**. Moreover, ICHs in thermal insulation Al tapes are capable of distinguishing various exercise states based on acceleration changes on the movable joint (yellow part). During walking, the presented green line exhibits a regular sine wave‐like waveform, corresponding to the gentle and slow arm swings. In contrast, jogging results in an irregular waveform with accelerated swing speeds and a more compact pattern. When at rest, the arm ceases swinging, leading to a straight‐line waveform. They agreed reasonably well with those from the Figure  4b. Importantly, in the event of circuit damage (e.g., a cut from a blade), the interrupted data can be restored once the device undergoes self‐healing. In practical applications, this capability allows the fractured surface of the device to quickly regain its intended properties and functionality, significantly enhancing mechanical robustness and prolonging the lifespan of the electronics.^[^ ^42^ ^]^ To demonstrate its long‐term stability for health monitoring, Figure  4f indicates that ICHs exhibited a reliable profile during 45 days, showing its promise as a wearable platform to reflect the health status of 3 different volunteers without crosstalk.^[^ ^47^ ^]^ To assess the skin compatibility of the ICHs, the hydrogel was attached to the forearm for 12 h (Figure S15d, Supporting Information). No inflammatory reactions or discomfort were observed during the entire period of contact. Additionally, cytotoxicity tests were performed using C2C12 cells for biocompatibility studies (Figure S15e, Supporting Information). The results indicated that the ICHs did not cause significant cell death or toxic effects after 24 h incubation, confirming their safety for long‐term skin contact. The slight variation in electrical signals for temperature monitoring and motion detection after 45 days (Figure S16, Supporting Information) was associated with a small amount of water evaporation in ICHs during this period. All human subject studies were approved by the Ethics Committee of the school of life science of technology, Xi'an Jiaotong University (protocol: (2024)78), and the volunteers gave informed consent. The authors affirm that human research participants provided informed consent for publication of the images.

## 3 Discussion

The integration of high‐conductivity, self‐healing, and adhesive ionic hydrogels in wearable sensors represents a transformative advancement in health monitoring technologies, particularly under extreme cold conditions.^[^ ^47a^ ^]^ These hydrogels possess a unique combination of mechanical flexibility and ionic conductivity, enabling real‐time physiological monitoring with high accuracy. This capability is crucial for applications such as remote health surveillance for outdoor workers, athletes, and individuals in cold climates, where environmental factors can significantly affect sensor performance.^[^ ^19^ ^]^

Self‐healing capabilities are paramount for enhancing the longevity and reliability of wearable devices.^[^ ^48^ ^]^ By incorporating dynamic cross‐linking networks, these hydrogels can autonomously repair micro‐damages caused by mechanical stress or environmental factors, thereby maintaining consistent functionality and reducing the need for frequent replacements. This aspect not only increases the lifespan of the devices but also minimizes electronic waste, aligning with sustainable practices in technology development.^[^ ^10d^ ^]^ The self‐healing ability of the ICHs designed in this study is less effective at −80 °C due to the slowed molecular and ionic mobility in the hydrogel matrix. At low temperatures, the reduced water content and freezing of water in the hydrogel hinder the efficiency of hydrogen bond formation and ionic interactions, which are key to the self‐healing process.^[^ ^19^ ^]^ To address this limitation, Adding anti‐freezing agents, such as glycerol or ionic liquids, could prevent water from freezing and help maintain the mobility of polymer chains at low temperatures.^[^ ^9a^ ^]^ Besides, we are considering introducing temperature‐responsive crosslinking or thermoreversible bonds, which could support self‐healing at lower temperatures by preserving flexibility while maintaining structural integrity.^[^ ^15^, ^49^ ^]^ In addition, increasing the hydrophilicity of the polymer backbone or utilizing more flexible polymers could help retain water content and improve self‐healing efficiency at low temperatures.^[^ ^50^ ^]^ Lastly, optimizing the healing time by introducing a multi‐phase healing process or using thermal activation could allow for gradual self‐healing, ensuring long‐term recovery even at low temperatures.^[^ ^51^ ^]^ These several approaches will be the main focus for enhancing the self‐healing performance of ICHs under extreme cold conditions. Adhesive properties are equally essential for ensuring that wearable sensors maintain intimate contact with the skin, especially in cold conditions where traditional adhesives may fail.^[^ ^44^ ^]^ The development of hydrogels that can adhere effectively to diverse skin types while maintaining comfort is key for user compliance and data integrity. Furthermore, the ability of these materials to function optimally at extreme low temperatures can mitigate risks associated with extreme low temperature‐induced sensor malfunctions, such as signal degradation or complete failure.^[^ ^52^ ^]^

While the self‐healing properties of the ICHs are promising, we acknowledge that prolonged mechanical cycling, such as repeated stretching or bending, may lead to cumulative damage that could affect the performance of the material over time. This issue is particularly relevant for wearable electronics, which are subject to constant movement and deformation.^[^ ^53^ ^]^ Although the self‐healing mechanism improves durability, we have highlighted the need for further studies to quantify the effects of mechanical fatigue on the conductivity, adhesion strength, and healing efficiency under repeated stress.^[^ ^41^ ^]^ Future work could explore strategies to enhance the fatigue resistance of the ICHs, potentially by optimizing the polymer network design or incorporating additional reinforcing agents. In addition, the scalability of ICHs for mass production is a critical consideration for their use in commercial wearable electronics.^[^ ^46^ ^]^ The preparation of ICHs, especially with the incorporation of components like TEMPO‐CNF and LiCl requires precise control over synthesis conditions. Variations in the concentration of these components or the gelation process could affect the uniformity and properties of the ICHs. For large‐scale manufacturing, achieving consistent material properties across batches is essential for reliable performance in end‐user applications. While the synthesis of ICHs in small quantities is feasible, scaling to industrial levels may require adapting the production methods to ensure cost‐effectiveness and process efficiency.^[^ ^3c^ ^]^ For example, large‐scale incorporation of CNFs into the polymer matrix could be challenging due to issues related to dispersion and uniform distribution.^[^ ^2a^ ^]^ Furthermore, the integration of ICHs into wearable devices (such as skin‐like patches or sensors) will require specialized fabrication techniques, such as roll‐to‐roll processing or 3D printing, which are still being optimized for soft, flexible materials.^[^ ^12^ ^]^ Besides, the materials used in ICHs, such as high‐purity CNFs and LiCl, may incur higher costs when produced in large quantities.^[^ ^54^ ^]^ The development of more affordable alternatives or optimization of material usage could play a pivotal role in making ICH‐based devices more commercially viable. Future research should concentrate on optimizing the formulation of these hydrogels to improve their performance in extreme cold conditions.^[^ ^55^ ^]^ Moreover, exploring the interplay between material properties and user comfort will be vital for achieving broader adoption in consumer markets.^[^ ^56^ ^]^

In this study, we demonstrate the potential of ICHs for a wide range of applications, including health monitoring and human‐machine interactions. While these applications are closely related within the context of wearable and interactive technologies. Health monitoring systems, such as wearable sensors designed to track physiological parameters (e.g., body temperature, heart rate, or muscle activity), inherently rely on human‐machine interactions to provide real‐time feedback, data analysis, and adaptive responses.^[^ ^57^ ^]^ This interaction allows health monitoring systems not only to gather data but also to communicate or act upon that data, enhancing user experience and engagement.^[^ ^3f^ ^]^ In this context, we position health monitoring as a subset of the broader human‐machine interactions domain, where ICHs serve as the critical material platform for integrating biological tissues with electronic systems.^[^ ^58^ ^]^ The unique properties of ICHs, including their high ionic conductivity, self‐healing ability, and strong adhesion under extreme conditions, make them ideal for both health monitoring and human‐machine interactions applications. In both cases, ICHs enable the seamless interaction between wearable devices and the user, ensuring that data collection and real‐time adaptive feedback occur reliably in challenging environments.^[^ ^59^ ^]^ By highlighting the relationship between health monitoring and human‐machine interactions, we underscore the versatility of ICHs as a material system for next‐generation wearable devices. These devices can provide continuous, real‐time monitoring while also allowing adaptive responses through interactive, intelligent interfaces.^[^ ^31^ ^]^

These ICHs could also be a potential alternative for being utilized in different aspects of applications. Due to their high flexibility, conductivity, and self‐healing capabilities, ICHs have significant potential in soft robotics.^[^ ^60^ ^]^ These hydrogels can be used as components in soft actuators or artificial muscles, where the ability to heal from mechanical damage and maintain stable performance under deformation is critical.^[^ ^41^ ^]^ Additionally, their ionic conductivity allows for responsive and adaptive behavior, making them ideal for responsive soft robotic systems.^[^ ^45^ ^]^ In aerospace applications, the ability of ICHs to operate effectively under extreme temperatures, including both high and low, makes them a strong candidate for use in spacecraft and satellites.^[^ ^54^ ^]^ Their durability, flexibility, and self‐healing properties can be beneficial in harsh environments, where traditional materials might degrade or fail due to thermal cycling or exposure to radiation.^[^ ^54^ ^]^ ICHs could be employed in sensors or flexible electronics used in aerospace systems that need to perform reliably under fluctuating and extreme conditions. In addition, the potential for ICHs in flexible electronics extends beyond wearable health monitoring to applications such as flexible displays, electronic skin, and stretchable batteries.^[^ ^29^, ^45^ ^]^ Their high conductivity and strong adhesion, even under low temperatures, position them as an ideal material for flexible electronics that require mechanical flexibility and long‐term stability in challenging environments.^[^ ^53^, ^61^ ^]^

In addition to the material properties and applications discussed, we believe that the integration of artificial intelligence (AI) techniques, particularly machine learning (ML) and predictive algorithms, holds tremendous potential for advancing the design and optimization of ICHs.^[^ ^62^ ^]^ AI‐driven simulations and data analysis could revolutionize the way hydrogels are developed by predicting material behavior, optimizing formulations, and accelerating the discovery of novel hydrogel systems.^[^ ^63^ ^]^ Machine learning models can be employed to analyze large datasets from experimental studies, identifying relationships between chemical composition, structure, and performance under various environmental conditions.^[^ ^64^ ^]^ By doing so, AI can aid in designing hydrogels with enhanced properties such as higher conductivity, greater self‐healing efficiency, and improved adhesion, especially in challenging environments. Moreover, predictive algorithms can be used to simulate the behavior of ICHs under a wide range of conditions, reducing the time and cost required for material testing and providing insights into long‐term stability and durability.^[^ ^65^ ^]^ This approach could accelerate the development of advanced hydrogels for diverse applications, from wearable health monitoring systems to flexible electronics and soft robotics.^[^ ^66^ ^]^ The integration of AI into hydrogel research represents a significant frontier in material science, where the fusion of experimental data with computational models could lead to a new era of smart materials that are not only highly functional but also adaptable to various environmental and operational demands.^[^ ^67^ ^]^ By incorporating AI tools into the development process, we can anticipate a future where ICHs and other hydrogels are tailored for optimal performance in real‐world applications, ensuring rapid advancements in health monitoring and human‐machine interactions.

## 4 Conclusion

In conclusion, the developed ICHs demonstrate exceptional conductivity (0.49 ± 0.05 S m^−1^), adhesion (36.73 ± 2.28 kPa), and self‐healing capabilities, even in extreme environments down to −80 °C. Their robust anti‐freezing properties and long‐term stability allow them for consistent performance over 45 days, addressing the significant challenge of gel‐based materials malfunctioning at sub‐zero temperatures. This multifunctionality provides a strong foundation for practical applications in health monitoring and human‐machine interactions under extreme conditions. By enabling high‐fidelity signal transmission, these ICHs overcome the limitations of conventional metallic wires, which are typically rigid and brittle, thereby facilitating the development of fully flexible and integrated systems. The conformity of the hydrogels allows them to strongly adhere to skin, ensuring non‐invasive, continuous health monitoring without signal crosstalk. This innovation paves the way for broader applications in smart healthcare and the Internet of Things, making ICHs a promising platform for future wearable devices and intelligent systems in harsh environment temperatures.

## 5 Experimental Section

### Materials

3‐[N,N‐dimethyl]‐[2‐(2‐methylprop‐2‐enoyloxy)ethyl]ammonium]propane‐1‐sulfonate inner salt (SBMA), N, N‐Methylenebisacrylamide (MBA), 2‐Methacrylic Acid (MAA), Sodium alginate (SA) were purchased from Alfa Aesar (China) Chemical Co., Ltd. Synthetic lithium silicate (Laponite) was purchased from NANOCOR, LLC. Tetramethylethylenediamine (TEMED) was purchased from Shanghai Meryer Biochemical Technology Co., Ltd. Cellulose nanofiber (Tempo‐CNF) was purchased from Tianjin Mujingling Biotech Co. Anhydrous lithium chloride (LiCl), Ammonium persulfate (APS) were purchased from Sigma‐Aldrich Corporation. All chemicals in experiments were used as supplied without any modification in experiments.

### Preparation of SBMA/MAA/SA Hydrogels

The SBMA/MAA/SA hydrogels were prepared by a one‐step method. Firstly, SBMA (6 g), MBA (0.12 g), SA (0.5 g), Laponite (0.018 g), MAA (4 mL), Tempo‐CNF (600 µL) and TEMED (40 µL) were dissolved in deionized water (10 mL), and the solution was vacuuming to remove air bubbles with continuously stirring at room temperature for 40 mins. Subsequently, LiCl (4.0 g) and APS (0.4 g) were dissolved in this solution and stirred for 10 min at room temperature. Then, the homogenized solution was shaped in a PMMA mold and cooled at room temperature over 24 h to obtain hydrogels. Finally, the hydrogels were sealed and stored at room temperature for further study.

### Differential Scanning Calorimeter (DSC)

The frost‐resistance property of the hydrogel is determined by a differential scanning calorimeter (DSC 250, Shaanxi Longrun International Trading Co., Ltd.). During the test, the samples were first equilibrated at −80 °C, and then the temperature increased at a rate of 10 °C min^−1^ until reaching 20 °C.

### Hydrogel Water Retention Test

Each sample was weighed and recorded as W_0_, and the weighed samples were stored at room temperature. After 1, 15, 30, and 45 days, the samples were weighed again to record as *W_t_*. The water retention of hydrogels was defined in the following equation:

(1) $$Weight\mathrm{loss}=\frac{W_{t}-W_{0}}{W_{0}}\times 100\%$$

### Mechanical Properties Measurement

The mechanical properties of hydrogels were measured by a tensile tester (TM2101‐T7, Dongguan Bolaide Instrument Co.) at room temperature with a speed of 100 mm min^−1^. Each sample was cut into a rectangular shape with a width of 12 mm, a height of 2 mm, and a gauge length of 30 mm. The tensile strength refers to the ratio of the force to the cross‐sectional area of the sample when it breaks. The tensile strength is calculated as follows:

(2) $$\sigma =\frac{F_{t}}{S_{o}}$$

*F_t_* represented the load of the hydrogel sample (N) and *S*_0_ represented the cross‐sectional area of the hydrogel sample before tensile testing (m^2^). Breaking elongation (Facture Strain) means the ratio of the max change in length when breaking to the initial length. The breaking elongation is calculated as follows:

(3) $$\sigma =\frac{L_{t}-L_{0}}{L_{0}}$$

*L_t_* represented the length of the hydrogel stretched to the moment t (mm), L_0_ represented the original length of the hydrogel sample without being stretched (mm).

### Adhesion Properties Measurement

The adhesiveness of hydrogels was carried out by a tensile tester (TM2101‐T7, Dongguan Bolaide Instrument Co.) at room temperature with a speed of 50 mm min^−1^. Glass, Rubber, Aluminum Foil, Copper Foil, and pig skin were chosen as the substrates for testing with hydrogels. The length, width, and height of test samples were 30 mm, 12 mm, and 2 mm, respectively. During the test, the hydrogel sample was fixed between the two substrates and pressed tightly against the substrates to obtain the test sample, and then the test sample was subjected to lap‐shear tensile testing until the hydrogel sample was completely detached from either of the substrates.

### Self‐Healing Measurement

The self‐healing properties of the hydrogel were measured by the tensile tester and digital universal meter. Briefly, the hydrogel was cut in half with a blade and then stuck together for 2 h. The tensile stress‐strain curve after healing was measured by the tensile tester when the samples were stuck together. Then, the hydrogel after healing with an LED light bulb connected in one circuit was used to test self‐healing properties of the hydrogel. The brightness of the bulb manifested the self‐healing properties of the hydrogel. The self‐healing properties were tested at −22 °C, room temperature, and −80 °C.

### Conductivity Measurement

The conductivity and resistance of hydrogel were measured using an electrochemical impedance spectroscopy (CHI650E, CH Instruments). The conductivity of hydrogels was calculated by the following equation:

(4) $$\sigma =\frac{L}{RS}$$

L (cm) represented the distance between every two electrodes, S (cm^2^) represented the cross‐sectional area of hydrogels and R (Ω) represented the hydrogel resistance.

### Stability Measurement

After 1, 15, 30, and 45 days of the hydrogels were made, hydrogel water retention test, tensile test, peeling‐off measurement, and conductivity measurements were conducted as the methods mentioned above to test the long time stability of the hydrogels.

### Information Transmission Test

Signal generator (sdg2042X, Ding Yang Technology Co., Ltd.) and oscilloscope (SDS6054, Ding Yang Technology Co., Ltd.) were used to set up an information transmission platform to test the information transmission performance of the hydrogel samples. Moreover, a robotic hand (Yhand advanced version, Hangzhou Songjia Technology Co., Ltd.) was used to establish a human‐machine interaction platform, which is able to transform the electrical signal of the ion‐conducting hydrogel samples into gestures of the robotic hand.

### Sensing Performance and Human Motion Detection

The electrical conductivity of hydrogel under strain was measured with the digital universal meter (34465A, Shidetech Technology Limited, China). In the sensing test, the hydrogel was molded into a rectangle with a thickness of 2 mm, a length of 20 mm and a width of 10 mm, which was attached to the throat, fingers, wrists, elbows, knees, and neck parts of a volunteer. Then the hydrogel sensor is connected to the digital universal meter.to record the real‐time electrical conductivity. The relative change of electrical conductivity is calculated as follows:

(5) $$\Delta R=\frac{R-R_{0}}{R_{0}}\times 100\%$$

𝑅_0_ represented the initial electrical conductivity and 𝑅 represented the real‐time electrical conductivity. The variations of the electrical signals at 4 °C, room temperature, and 40 °C were all tested by the digital universal meter.

### Thermosensitive Performance Assessment

For the accurate determination of the temperature, the experimental data was recorded after the current curve fluctuation became stabilized. To evaluate the ICHs for temperature monitoring, the electronic thermometer and infrared (IR) camera (ST9450) were used to detect the temperature change of the hydrogel and the temperature coefficient of resistance (TCR) was defined as:

(6) $$TCR=\frac{R_{T}-R_{0}}{R_{0}\times \Delta T}$$

where R_T_, R_0_, and ΔT represent instantaneous resistance, resistance at the initial temperature, and temperature change, respectively. All human subject studies were approved by the Ethics Committee of the school of life science of technology, Xi'an Jiaotong University (protocol: (2024)78), and the volunteers gave informed consent. The authors affirm that human research participants provided informed consent for publication of the images.

### Rheology Measurement

Rheology measurements of the ICHs were conducted with RheoWin MARS 40 Rheometer (Thermo Fisher Scientific) using a parallel plate of diameter 20 mm. The frequency sweep was performed over the range of 0–20 Hz at a fixed strain of 1%. All measurements were performed at room temperature.

### SAXS Measurement

The micromorphology of ICHs was analyzed by SAXS (SAXSpoint 2.0, Austria). The MetalJet X‐ray source (Excillum D2+) with a liquid metal anode was operated at 70 kV and 3.57 mA, emitting Ga‐Kα radiation with a wavelength of λ = 1.314 Å. The sample‐to‐detector distances ranged from 0.5 to 1.7 m. The effective scattering vector range q was from 0.03 to 1 nm^−1^ (q = 4Π sin θ/λ, where 2θ is the scattering angle and λ is the wavelength).

### SEM Measurement

The morphology and chemical structures of the hydrogels were measured by a scanning electron microscopy (HELIOS NanoLab 600i SEM, Thermo Fisher Scientific) in the working distance of 8 mm with an acceleration voltage of 15 kV. Before the measurement, the freezing‐dried hydrogel samples were respectively processed with gold to obtain high‐quality surface topography images.

### DRS Measurement

The samples were performed on a Novocontrol Concept 80 broadband dielectric spectrometer with temperature control. Each sample was deposited on the dielectric electrodes with a Teflon spacer of 0.1 mm. The sample diameter was 10 × 10 mm.

### In Vitro Cell Experiment

These were performed as follows: the 10 × 10 mm ICHs were sterilized by autoclaving at 121 °C for 20 min, and placed in a 24‐well plate, and C2C12 cells were seeded on the surface of ICHs. The cells were incubated for 24 h at 37 °C in a 5% CO_2_ incubator. Cells in 24‐well plates were washed with PBS to remove excess serum. The staining solution (Beyotime Biotechnology, China) was added for cell staining. Then the cells were observed and photographed using a fluorescence microscope (Olympus FV3000).

## Conflict of Interest

The authors declare no conflict of interest.

## Supporting information



Supporting Information

## Data Availability

The data that support the findings of this study are available from the corresponding author upon reasonable request.
