# Supplementary material for: High‐Conductivity, Self‐Healing, and Adhesive Ionic Hydrogels for Health Monitoring and Human‐Machine Interactions Under Extreme Cold Conditions
Source: Adv Sci (Weinh). 2025 Jan 28;12(16):2412726. doi: 10.1002/advs.202412726 (PMC12021042; doi:10.1002/advs.202412726)
Supplement: Supplementary file 1 — Supporting Information [file ADVS-12-2412726-s001.docx]

**Supporting Information**

**High-Conductivity, Self-Healing, and Adhesive Ionic Hydrogels for Health Monitoring and Human-Machine Interactions under Extreme Cold Conditions**

Fei Han^a,b#^ Shumeng Chen^a,b#^, Fei Wang^a,b^, Mei Liu^a,b^, Jiahui Li^a,b^, Hao Liu^a,b^, Yanshen Yang^a,b^, Haoqing Zhang^a,b^, Dong Liu^c^, Rongyan He^b,d^, Wentao Cao^a,b^, Xiaochuan Qin^a,b^, Feng Xu^a,b*^

*^a^ The Key Laboratory of Biomedical Information Engineering of Ministry of Education, School of Life Science and Technology, Xi’an Jiaotong University, Xi’an 710049, P.R. China*

*^b^ Bioinspired Engineering and Biomechanics Center (BEBC), Xi’an Jiaotong University, Xi’an 710049, P.R. China*

*^c^ School of Chemical Engineering and Technology, Xi’an Jiaotong University, No. 28, Xianning West Road, Xi’an, Shaanxi 710049, P. R. China*

*^d^ Guangxi Key Laboratory of Special Biomedicine, School of Medicine, Guangxi University, Nanning 530004, P. R. China*

*^#^ These authors contributed equally to this work.*

*^*^ Corresponding author: fengxu@mail.xjtu.edu.cn*

**
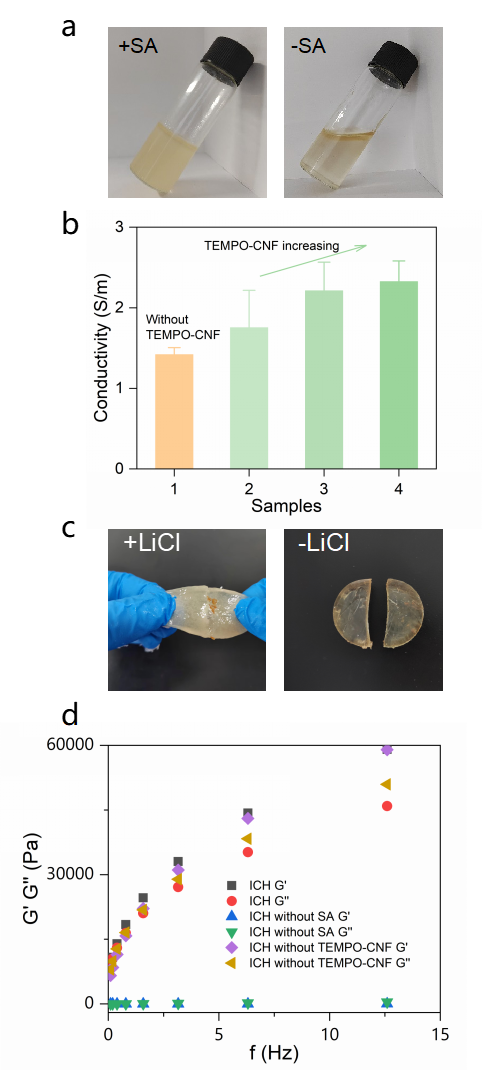
Figure S1.** (a) ICHs with and without addition of SA. (b) The conducitivity of ICHs with and without addition of TEMPO-CNF. (c) Self-healing of ICHs with and without addition of LiCl. (d) Rheology measurements of ICHs, ICHs without SA, and ICHs without TEMPO-CNF.

**
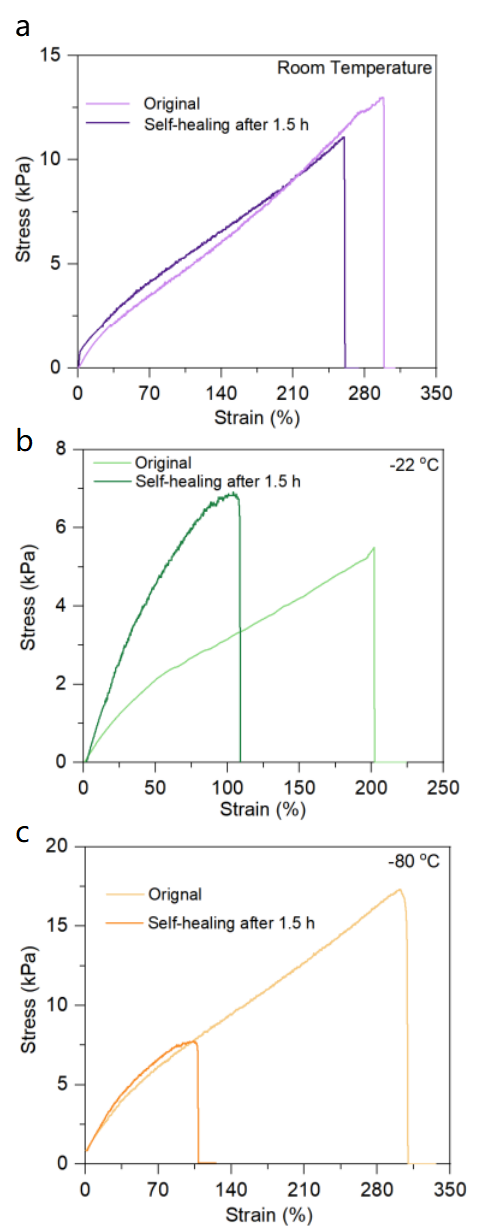
Figure S2.** The stress-strain curves of the corresponding self-healing efficiency in Figure 2c.


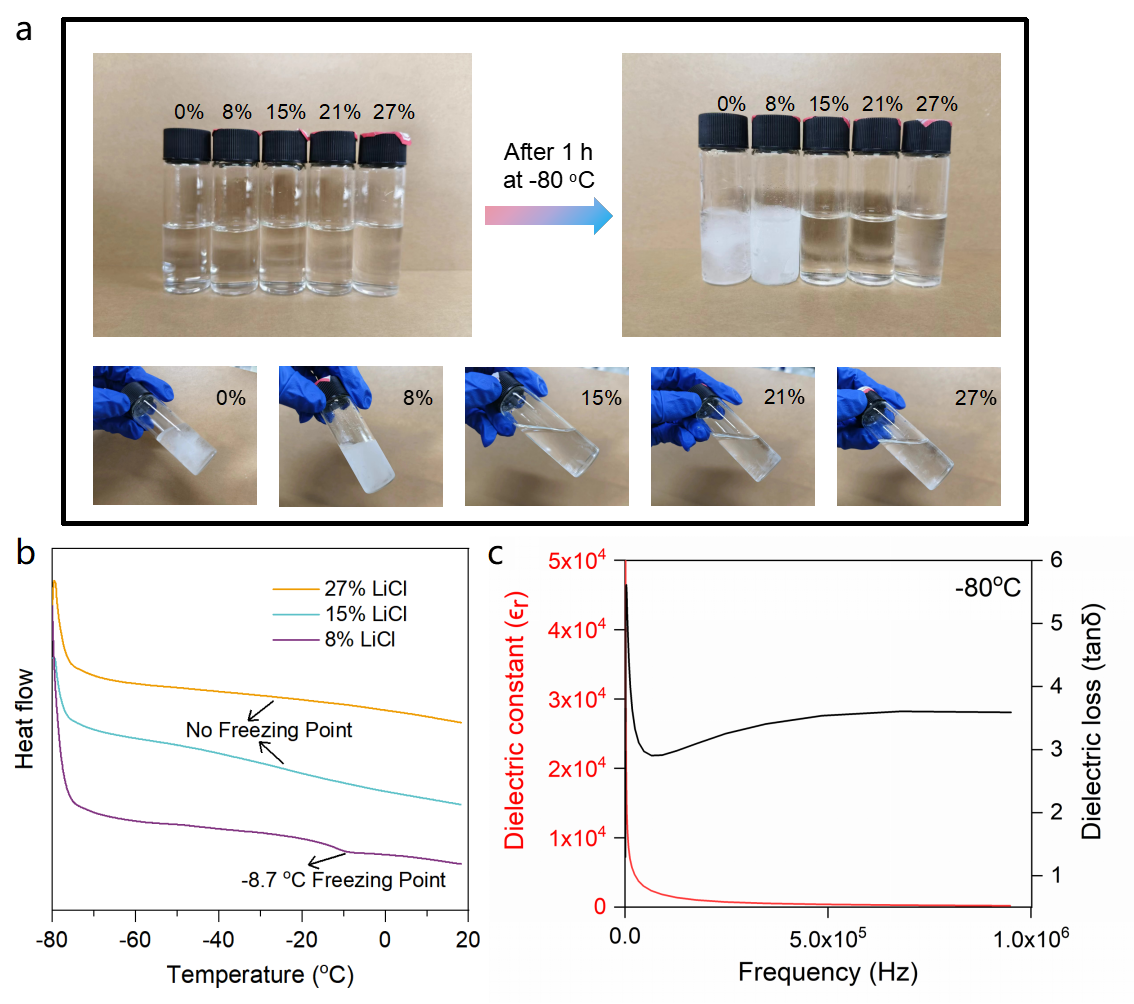


**Figure S3.** (a) The anti-freezing performance of four types of colligative inorganic salt after being stored in -80 ℃ for 1 h. There is no freezing phenomenon in the solution and gel of LiCl when its concentration is above 15%. (b) DSC tests of ICHs with different content of LiCl. (c) DRS measurement of ICHs under -80℃.


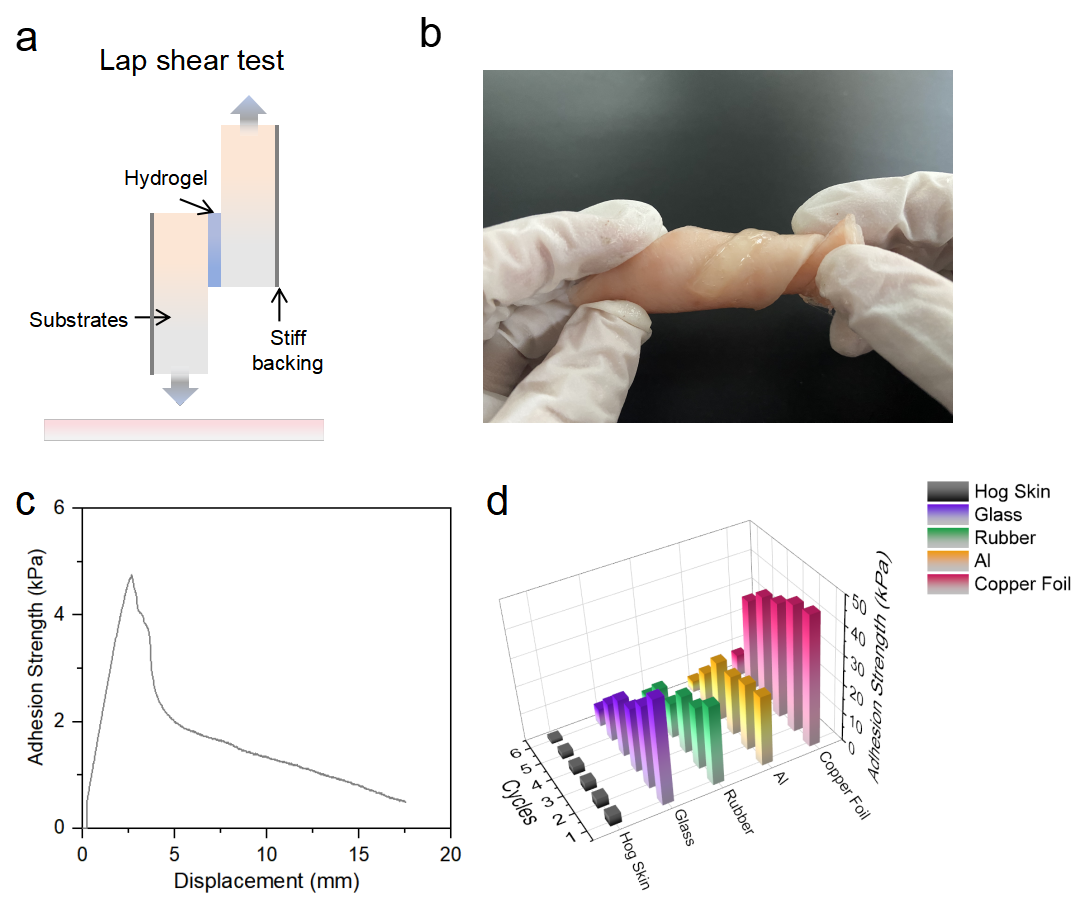


**Figure S4.** (a) Schematic representation of lap shear test geometry. (b) ICHs can be easily attached on hog skin. (c) Representative adhesion strength versus displacement curves of ICHs on hog skin. (d) The repeatable self-adhesion to various substrates (hog skin, glass, rubber, Al, copper foil) for 5 continuous attachment/detachment cycles.

**
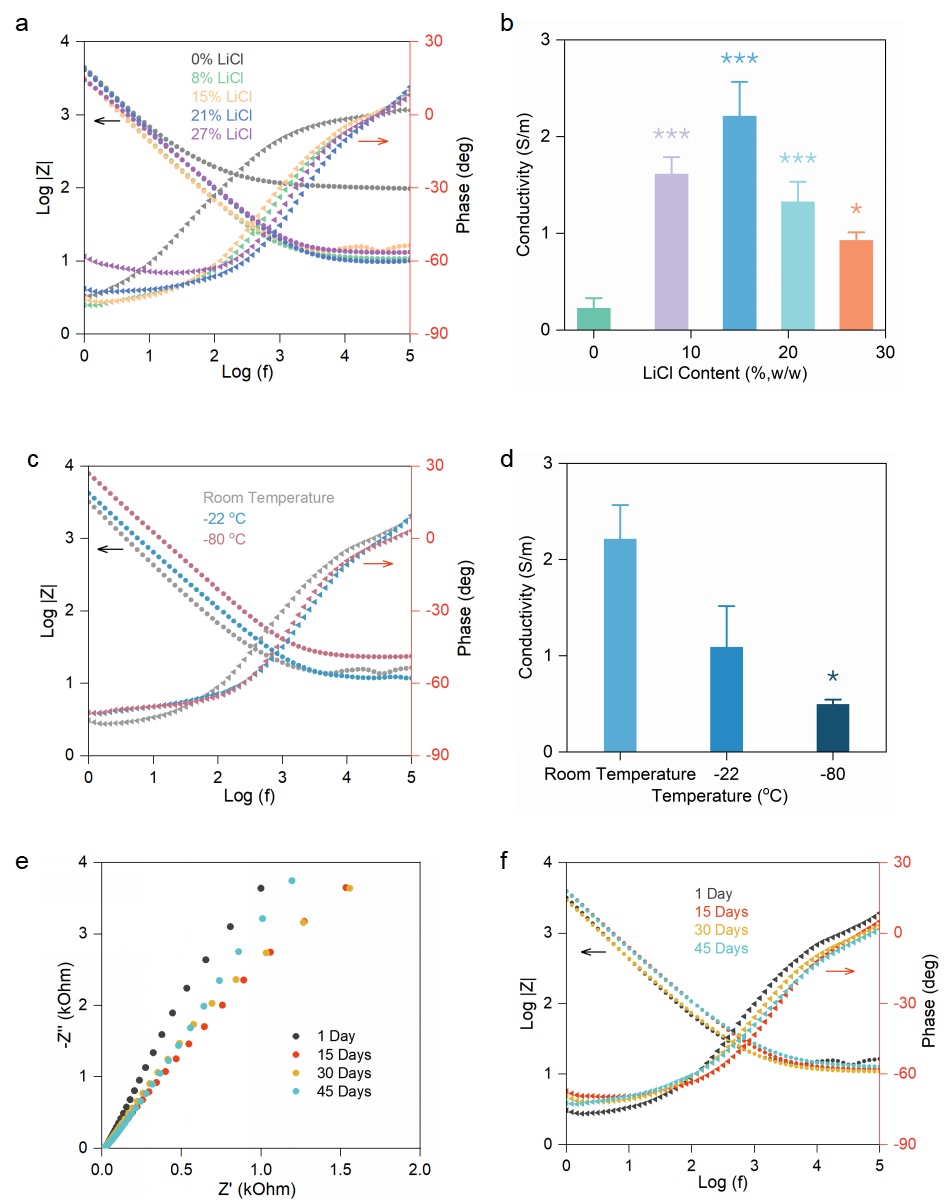
Figure S5.** (a) The impedance and phase plot (b) Conductivity of ICHs with different content of LiCl. *represent significant differences versus ICH, (c) The impedance and phase plot (d) conductivity of ICHs under different temperatures (room temperature, -22 and -80 ℃). *represent significant differences versus ICH. (e) EIS plot of ICHs under different storage times. (f)The impedance and phase plot of ICHs under different storage times. *p < 0.05, **p < 0.01, ***p < 0.001.


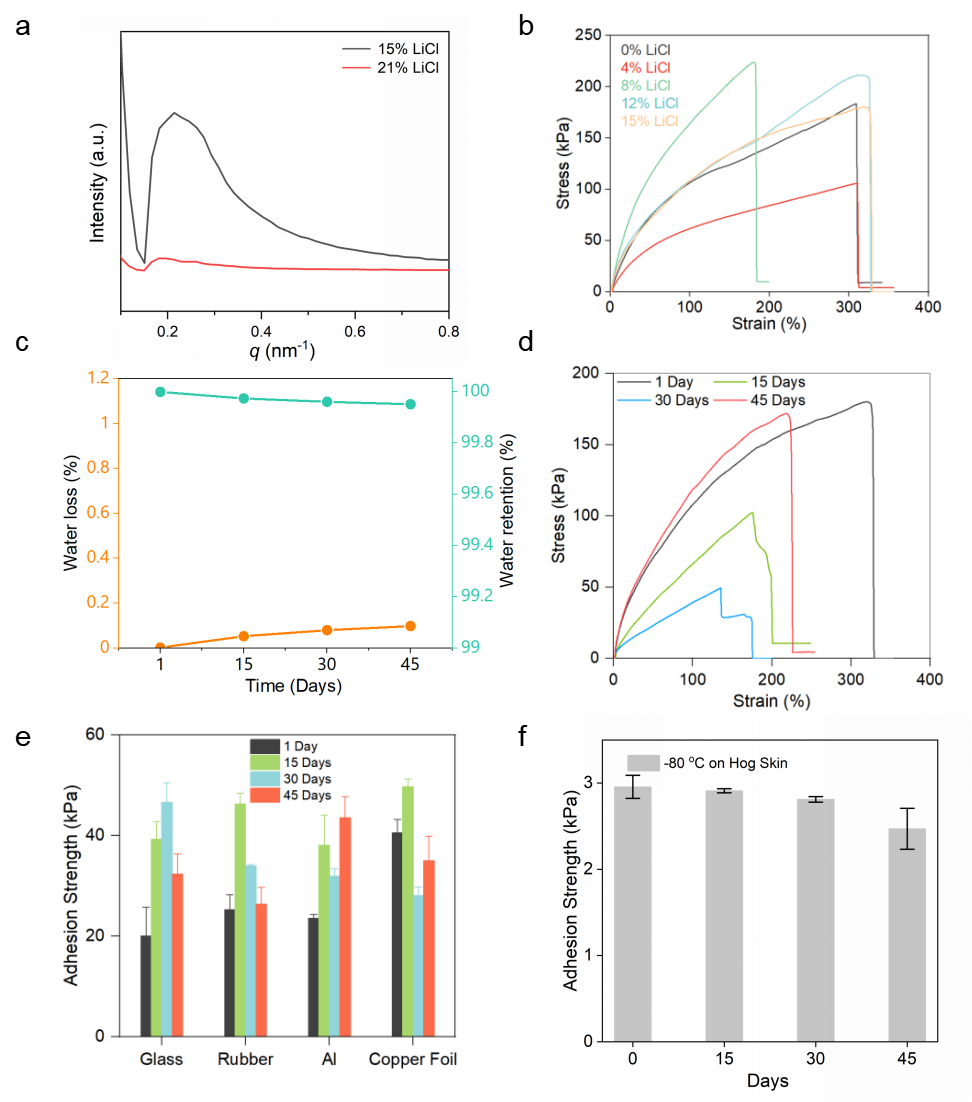


**Figure S6.** (a) The SAXS data of ICHs with 15% and 21% LiCl. (b) The stress-strain curves of ICHs with different content of LiCl. (c) Water loss and water retention of ICHs for 45 days. (d) The stress-strain curves of ICHs under different storage times. (e) The adhesion strength of ICHs under different storage times. (f) The adhesion properties of ICHs under -80℃ for 45 days.


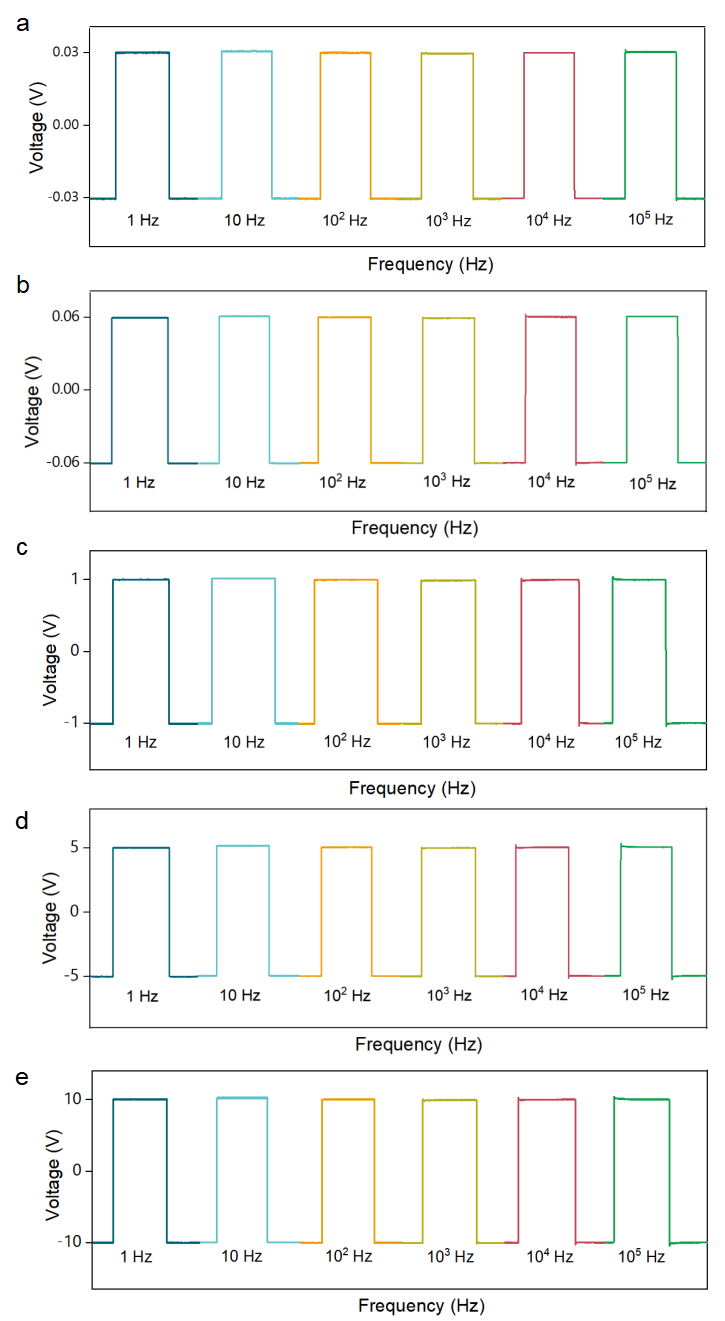


**Figure S7.** Signal transmission of ICHs in rectangular wave in a) ±0.03 V, b) ±0.06 V c) ±1 V, d) ±5 V e) ±10 V under frequency from 1 Hz to 10^5^ Hz.


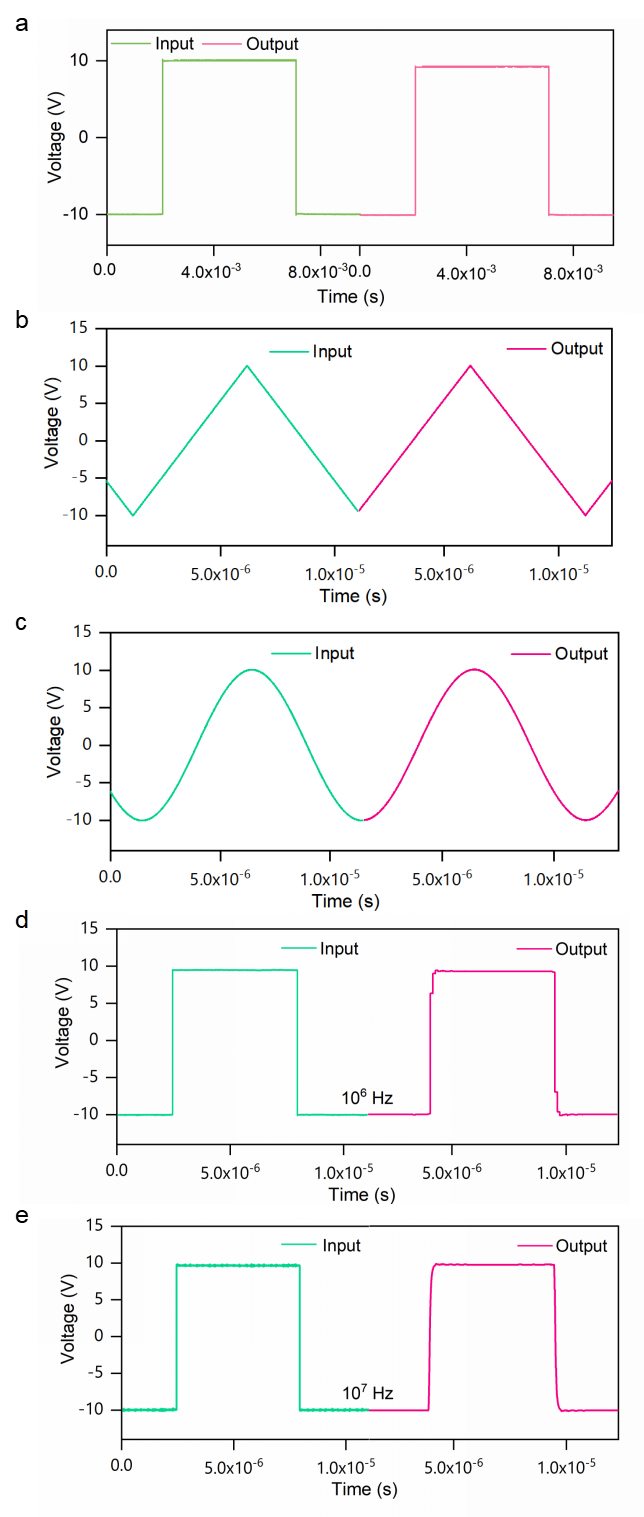


**Figure S8.** Signal transmission of ICHs in a) square wave, b) triangle wave, and c) sin wave. (d and e) Signal transmission of ICHs in rectangular wave under frequency from 10^6^ Hz to 10^7^ Hz.


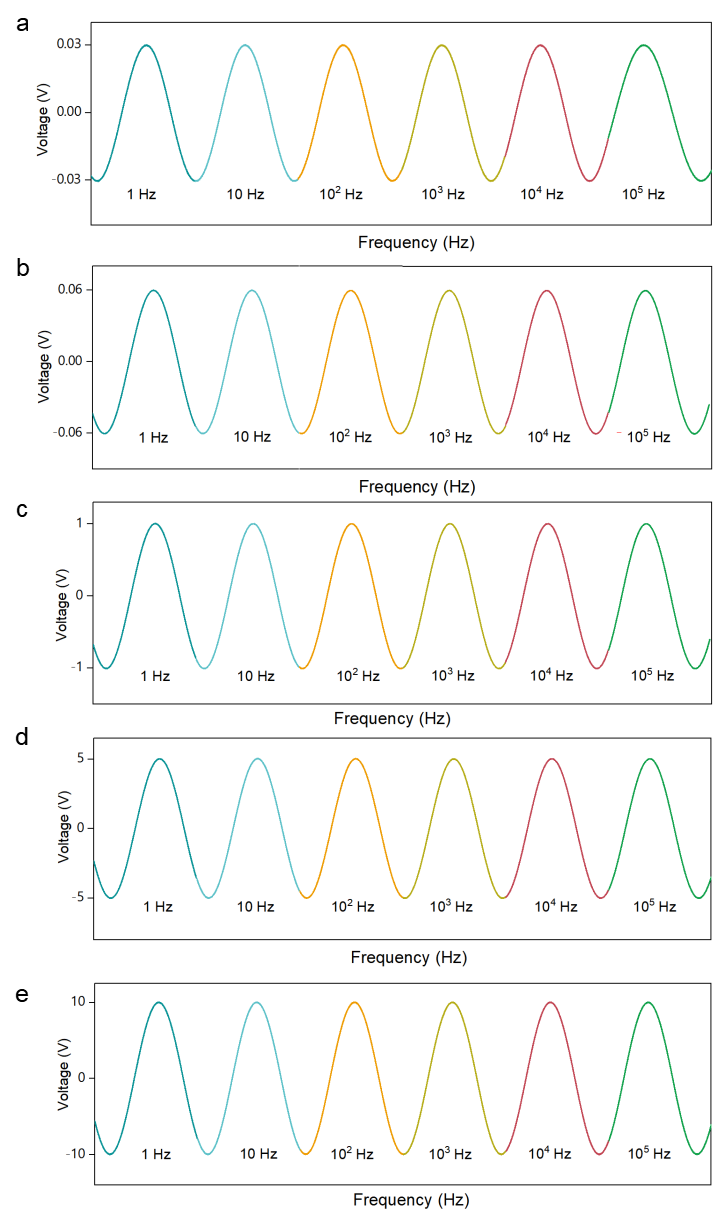


**Figure S9.** Signal transmission of ICHs in sin wave in a) ±0.03 V, b) ±0.06 V c) ±1 V, d) ±5 V e) ±10 V under frequency from 1 Hz to 10^5^ Hz.


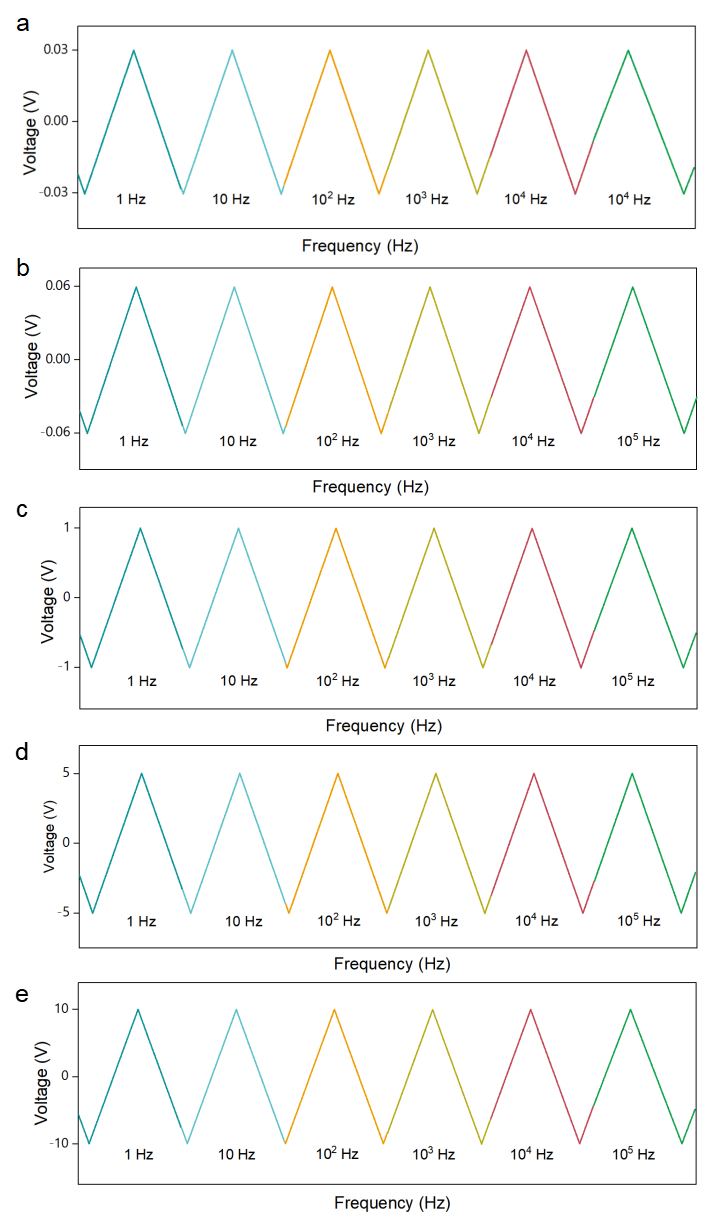


**Figure S10.** Signal transmission of ICHs in sin wave in a) ±0.03 V, b) ±0.06 V c) ±1 V, d) ±5 V e) ±10 V under frequency from 1 Hz to 10^5^ Hz.


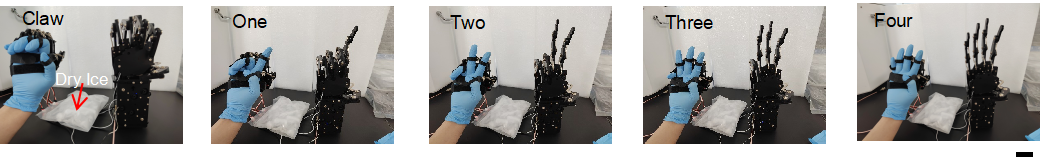


**Figure S11.** Stomatosensory glove and robotic hand connected by ICHs demonstrating different gestures at extreme low temperatures. Scale bar: 2 cm.

**
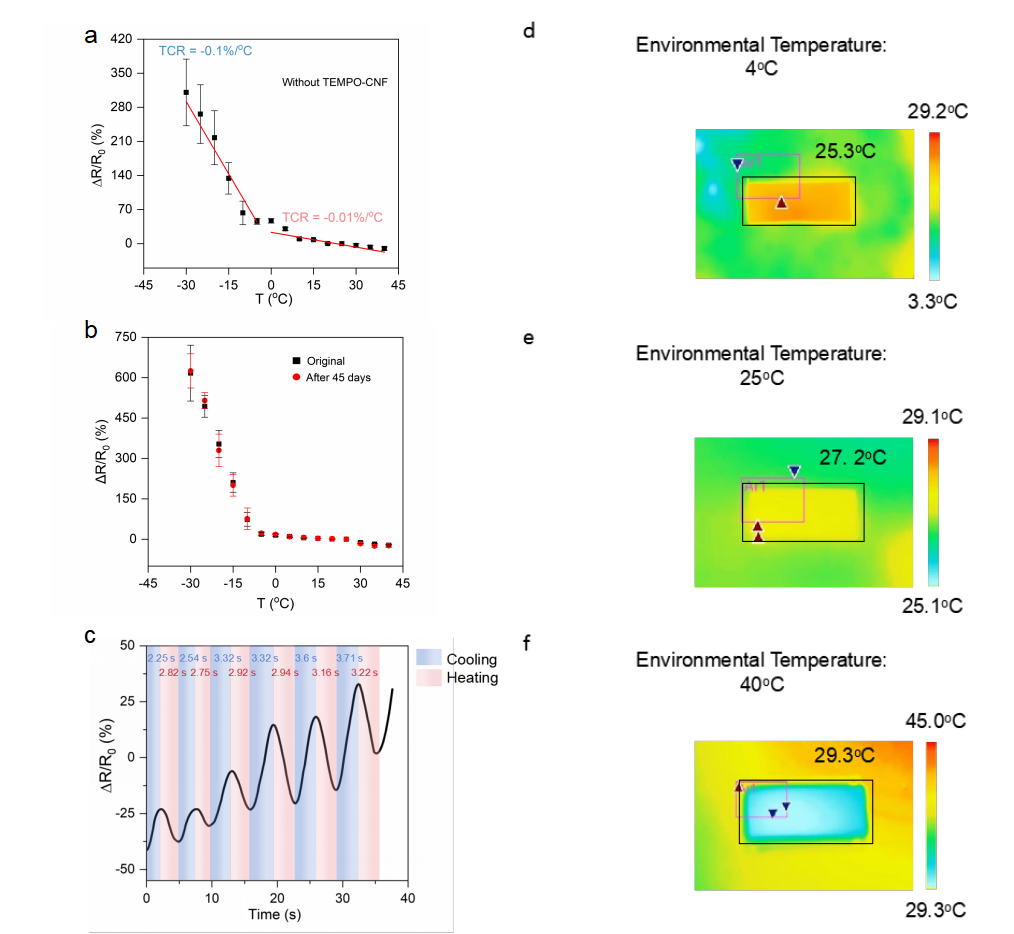
**

**Figure S12.** (a) Relative resistance variation of ICHs without TEMPO-CNF for temperature monitoring from -30 to 40 °C. The slope of the linear fitting curves demonstrates the TCR value of ICHs without TEMPO-CNF for temperature monitoring. (b) Stability measurements of TCR in ICHs after 45 days. (c) ΔR/R_0_ response of ICHs in a cyclic test of temperature after heating and cooling cycles. Thermal infrared images of ICHs in thermal insulation aluminium tapes under environmental temperatures: d) 4℃, e) 25℃, f) 40℃.


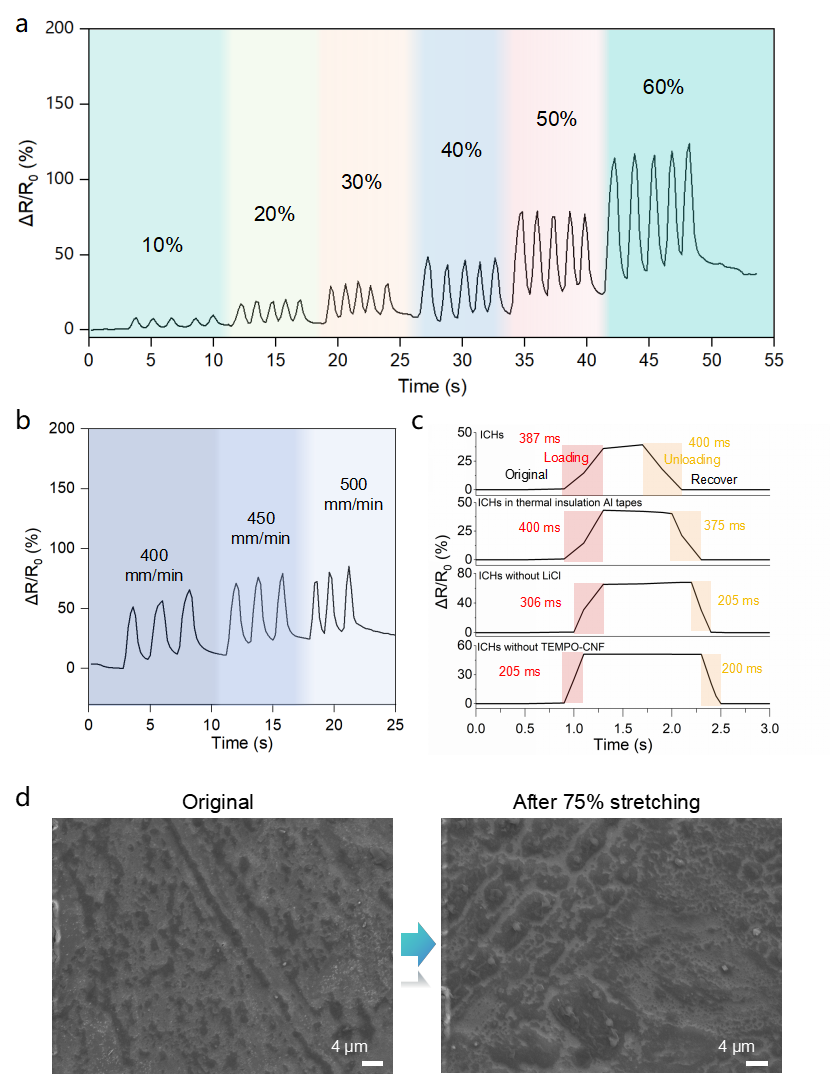


**Figure S13.** a) Time-dependent relative resistance changes of ICHs with different tensile strains. b) Relative resistance change under cyclic stretching-releasing tests at 40% strain with different stretching rates. c) Responsive time during tensile stretching and releasing process at 30% strain. d) SEM images of ICHs at original state and after 75% stretching.


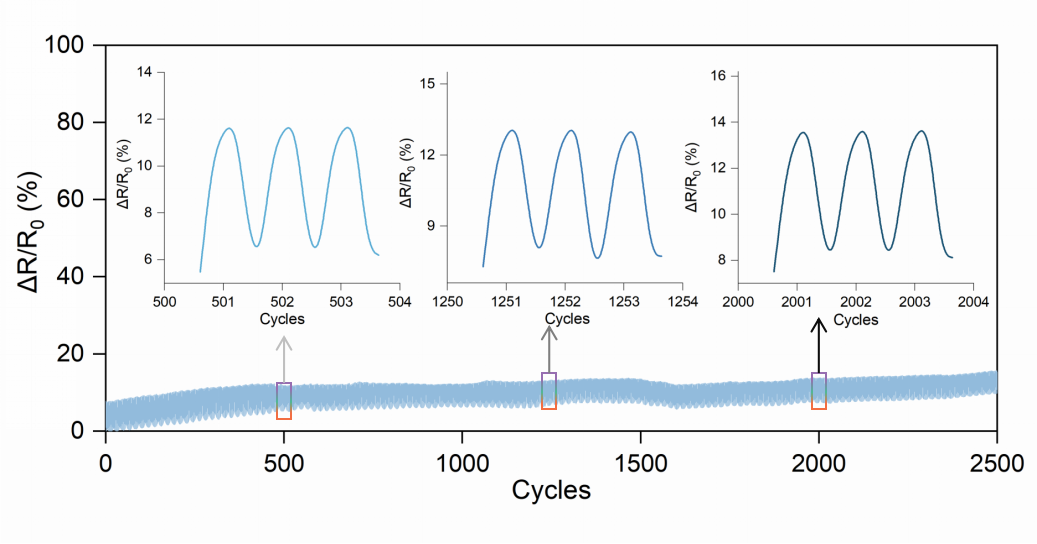


**Figure S14.** Relative resistance change under cyclic stretching-releasing tests for up to 2500 cycles at 10% tensile strain.


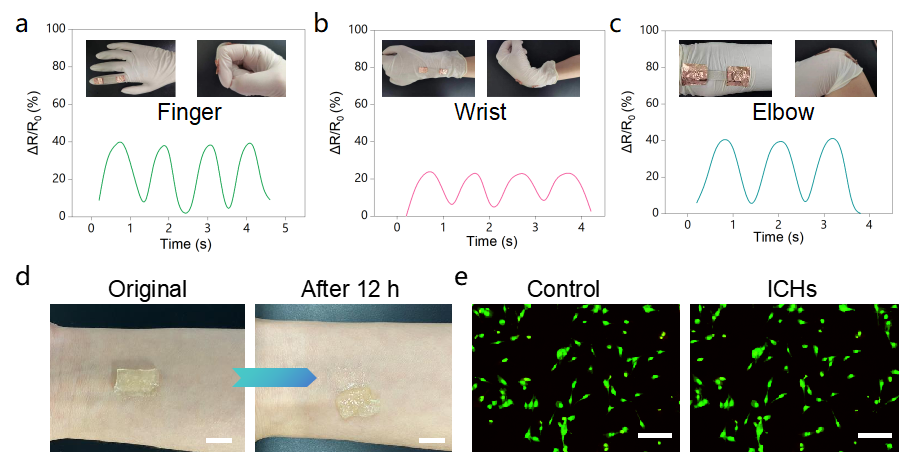


**Figure S15.** ICHs in thermal insulation Al tapes as a flexible sensor to monitor human motion. a) Finger flexion at 4℃. b) Wrist flexion at 25℃. c) Elbow bending under 40℃. d) Optical photographs of skin when the ICHs was attached after 12 h and then detached. e) The fluorescence staining images of C2C12 cells without and with ICHs after 24 h of culturing.


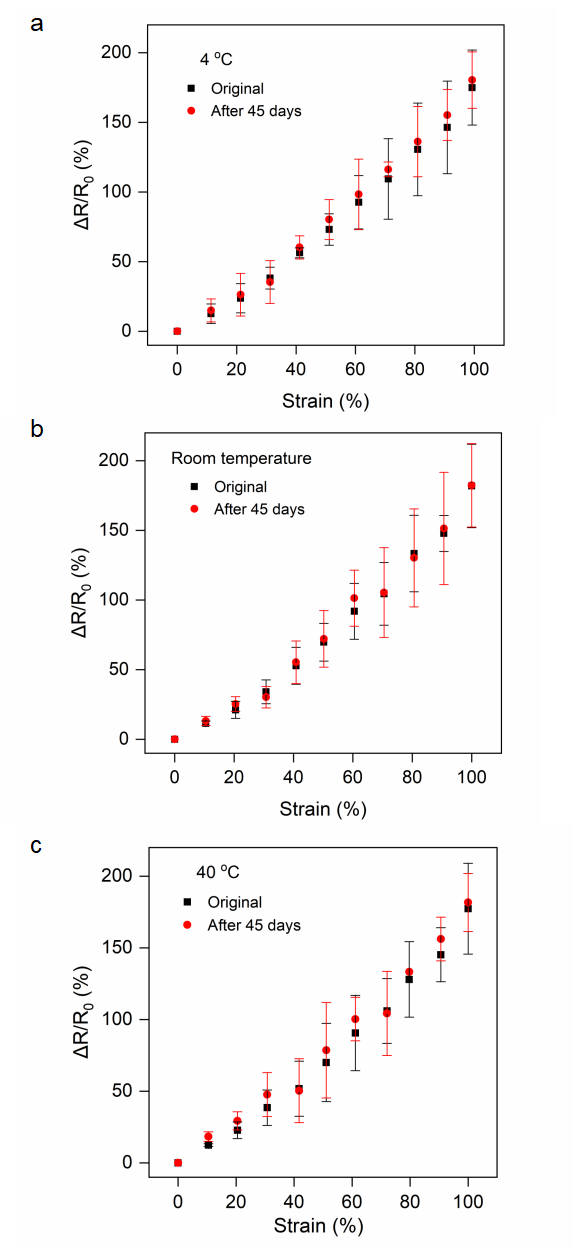
F**igure S16.** Long-term stability measurements of ICHs in thermal insulation Al tapes as a flexible sensor to monitor human motion. a) Finger flexion at 4℃. b) Wrist flexion at 25℃. c) Elbow bending under 40℃.

**Table S1.** ICHs with different healing efficiency under different conditions.

| Chemicals | weight (g) | Healed strain | Original Strain | Healing Efficiency |
| --- | --- | --- | --- | --- |
| SBMA | 3 | / | / | / |
|  | 6 | 158.37% | 447.97% | 35.35% |
|  | 9 | 67.58% | 434.24% | 15.56% |
| MBA | 0.06 | 183.33% | 486.32% | 37.70% |
|  | 0.12 | 158.37% | 447.97% | 35.35% |
|  | 0.24 | 42.49% | 239.69% | 17.73% |
| Laponite | 0.008 | 82.84% | 547.38% | 15.13% |
|  | 0.018 | 158.37% | 447.97% | 35.35% |
|  | 0.027 | 373.37% | 730.30% | 51.13% |
|  | 0.032 | 110.79% | 440.12% | 25.17% |
|  | 0.036 | 20.11% | 236.10% | 8.52% |
| SA | 0.25 | 110.47% | 410.15% | 26.93% |
|  | 0.5 | 158.37% | 447.97% | 35.35% |
|  | 1 | 76.46% | 372.17% | 20.54% |
| MAA | 2 | 28.15% | 186.34% | 15.11% |
|  | 4 | 158.37% | 447.97% | 35.35% |
|  | 6 | 179.84% | 579.24% | 31.05% |
| Tempo-CNF | 0.3 | 74.39% | 230.60% | 32.26% |
|  | 0.6 | 158.37% | 447.97% | 35.35% |
|  | 0.9 | 142.48% | 332.19% | 42.89% |
| TEMED | 0.02 | 342.25% | 727.42% | 47.05% |
|  | 0.04 | 158.37% | 447.97% | 35.35% |
|  | 0.06 | 87.07% | 302.30% | 28.80% |
| APS | 0.2 | 260.55% | 320.22% | 81.37% |
|  | 0.4 | 158.37% | 447.97% | 35.35% |
|  | 0.6 | / | / | / |
